# Comparative analysis of targeted metabolomic profiles reveals plasma metabolite differences across three Italian heavy pig breeds

**DOI:** 10.1038/s41598-025-23058-z

**Published:** 2025-11-10

**Authors:** Samuele Bovo, Matteo Bolner, Giuseppina Schiavo, Flaminia Fanelli, Giuliano Galimberti, Francesca Bertolini, Anisa Ribani, Stefania Dall’Olio, Paolo Zambonelli, Uberto Pagotto, Luca Fontanesi

**Affiliations:** 1 Animal and Food Genomics Group, Division of Animal Sciences, Department of Agricultural and Food Sciences, University of Bologna, Viale Giuseppe Fanin 46, Bologna, 40127 Italy; 2 Department of Surgical and Medical Sciences, Endocrinology Unit, University of Bologna, Via Giuseppe Massarenti 9, Bologna, 40138 Italy; 3 Department of Statistical Sciences “Paolo Fortunati”, University of Bologna, Via delle Belle Arti 41, Bologna, 40126 Italy

**Keywords:** Blood, Lipid metabolism, Machine learning, Metabolite, Metabolomics, *Sus scrofa*, Biochemistry, Biological techniques, Chemical biology, Genetics, Molecular biology

## Abstract

In livestock, many economically important traits vary among breeds and lines, reflecting the influence of different genetic backgrounds on trait expression. In this study, we assessed how molecular phenotypes vary among three heavy pig breeds, by analysing the level of more than 180 plasma metabolites using a targeted metabolomic platform. Plasma was from 12 Italian Large White, 12 Italian Duroc and 12 Italian Landrace pigs, raised and fed in the same way. Advanced data analysis methods, including the Boruta algorithm and random forest, were employed to compare plasma profiles in the three breeds. The level of 11, 14 and five metabolites differed between Italian Duroc and Italian Large White breeds, Italian Duroc and Italian Landrace breeds and Italian Large White and Italian Landrace breeds, respectively. Distinct breed-related metabolic profiles emerged, aligning with known characteristics of the breeds. Specifically, Italian Duroc pigs are marked by diminished biogenic amine levels, Italian Large White pigs by lower sphingomyelin levels, and Italian Landrace by elevated acylcarnitine and phosphatidylcholine levels. These results suggest potential differences in breed-related energy and lipid metabolism strategies. These findings could be used to develop new molecular phenotypes useful in pig breeding and selection and to explore breed-precision feeding strategies.

**Supplementary Information:**

The online version contains supplementary material available at 10.1038/s41598-025-23058-z.

## Introduction

Metabolomics is an omics discipline that studies the metabolome, defined as the full set of small molecules (metabolites) within a biological system^1,2^. Metabolites, as intermediate or final products of metabolic pathways, constitute molecular phenotypes that play crucial roles in fundamental biological processes. Therefore, metabolites serve as intermediaries linking the genetic background of an organism to more complex phenotypes^3^, making them valuable targets for identifying biomarkers associated with economically relevant traits in livestock species^4^. Given the complexity of datasets generated by metabolomic technologies such as mass spectrometry (MS) and nuclear magnetic resonance (NMR), appropriate statistical tools, including supervised and unsupervised models, are essential for accurate data analysis and interpretation^5,6^.

In pigs, many economically important traits, such as muscle mass, carcass composition, growth rate, feed efficiency, and reproductive performance vary among breeds and lines, reflecting the influence of different genetic backgrounds on trait expression^7,8^. These traits, seen as endpoint or final phenotypes, are shaped by complex biological processes, the underlying mechanisms of which remain largely unexplored^9^.

Comparative metabolomics between breeds assumes that genetic differences are the primary source of metabolic variation between breeds, when environmental factors are controlled^10^. Although this approach does not elucidate specific mechanisms, it provides initial evidence indirectly linking genotypes to metabolite levels. Identifying metabolic markers that differentiate breeds offers novel insights into breed-related and breed-specific physiological mechanisms governing breed differences in economically relevant traits. This supports the biological dissection of complex traits, with implications for understanding heterosis in pig crossbreeding programs^10^.

Several studies have explored breed-related metabolomic differences, although environmental confounding remains a challenge to really assume that breed-derived genetic diversity can be considered the factors determining claimed differences. For instance, a few studies identified muscle metabolic biomarkers distinguishing different breeds or crossbred pigs some of which associated with meat quality characteristics^11–16^. Rohart et al.^17^ utilized plasma metabolomic data to predict performance traits among Large White, Landrace, and Pietrain pigs. Additionally, He et al.^18^ compared serum metabolomes of obese Ningxiang pigs and lean Duroc × Landrace × Yorkshire crossbreds as a model for childhood obesity research.

We previously characterized plasma and serum metabolomes of Italian Large White and Italian Duroc pigs using targeted and untargeted approaches, and integrated these data with genomic information^10,19,20^. Building on this work, we now extend for the first time metabolomics analysis to include the Italian Landrace breed, a population of major economic relevance^21^ but not yet studied at the metabolomic level. Using a targeted mass spectrometry-based platform detecting approximately 180 metabolites, together with advanced data analysis methods we previously developed (Boruta algorithm and random forest^19,22^, we compared plasma profiles across the three heavy pig breeds: Italian Large White, Italian Duroc, and Italian Landrace. These three pig breeds, essential in producing three-way crossbred pigs used within the Italian heavy pig production system, exhibit significant differences in production traits, with Italian Duroc pigs having higher intramuscular and intermuscular fat content compared to both Italian Large White and Italian Landrace breeds. These breeds are similar to the corresponding cosmopolitan breeds. At the same time, we aim at confirming and extending previous findings obtained for the Italian Duroc and Italian Large White comparisons^10,19^, thus reinforcing the robustness and reproducibility of metabolomics approaches.

## Methods

**Fig. 1:**
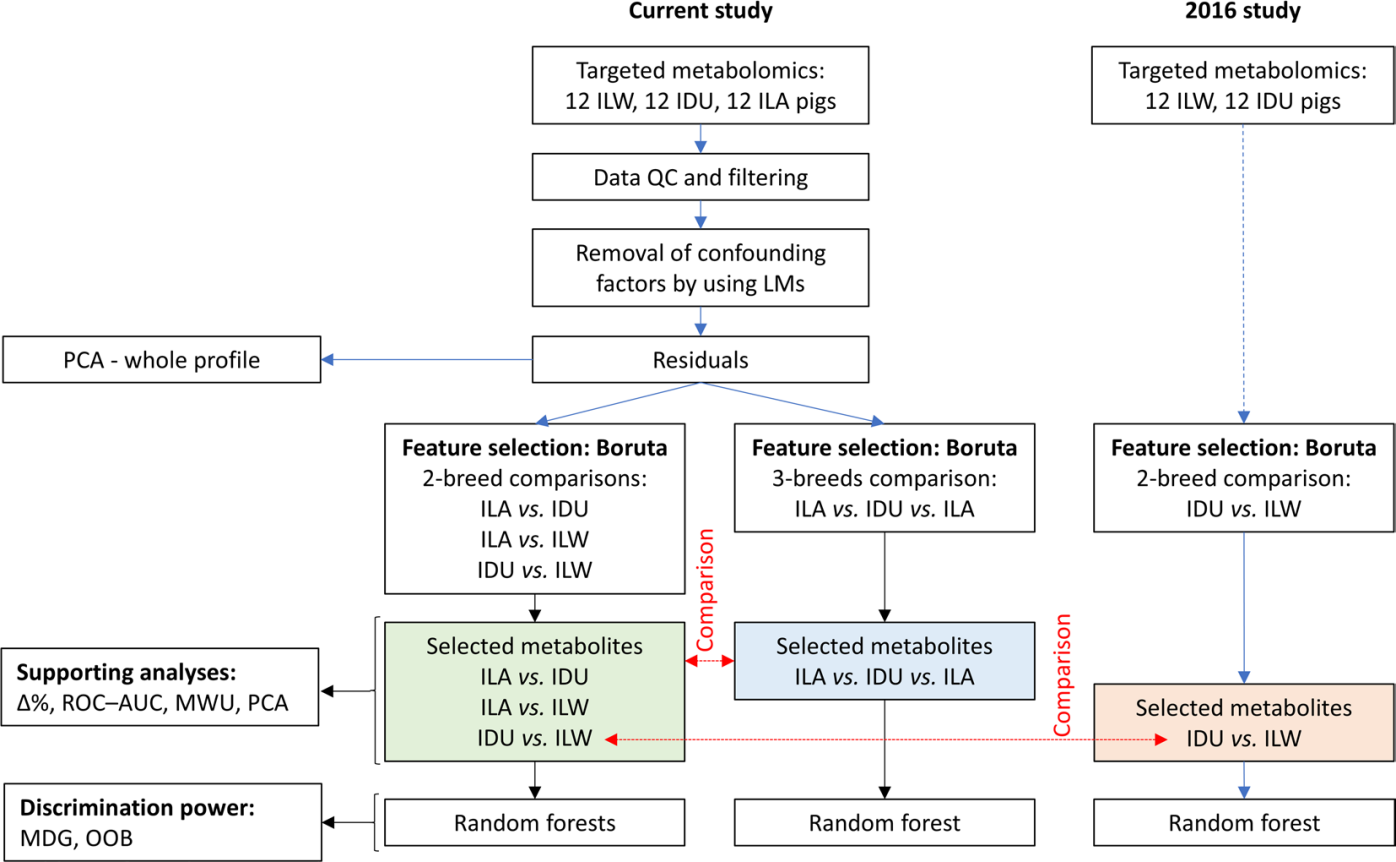
Overview of the analytical workflow. Abbreviations: ILW, Italian Large White; IDU, Italian Duroc; ILA, Italian Landrace; PCA, Principal Component Analysis; ROC-AUC, Receiver Operating Characteristic curve and Area Under the Curve; MWU, Mann-Whitney U test; MDG, Mean Decrease Gini; OOB, Out-Of-Bag; LMs, Linear Models; QC, Quality Control; Δ%, relative concentration difference between breeds.

Considering the range of analyses performed that will be detailed in the next sections, we provide here a brief overview of the main data analysis steps (Fig. 1): (1) plasma samples from pigs representing three Italian breeds were subjected to targeted metabolomics; (2) obtained data underwent quality control and were corrected for sex and carcass weight using linear regression; residuals were retained for subsequent analyses; (3) breed-related metabolites were identified through Boruta feature selection, applied both to pairwise breed comparisons and to the joint dataset including all three breeds; (4) selected metabolites were further evaluated using a random forest to obtain variable importance measures and classification errors; (5) the predictive performance of discriminant metabolites was assessed using Receiver Operating Characteristic (ROC) curve analysis and the area under the curve (AUC) and Mann-Whitney U tests (MWU); (6) Principal Component Analysis (PCA) was applied to investigate breed separation before and after feature selection; (7) finally, a previously generated metabolomics dataset (IDU and ILW) was reanalysed with the Boruta-based pipeline to validate earlier results and harmonize findings across studies. A complete description of the animals included in the study, the metabolomic platform used and the applied methodological steps is reported in the subsequent paragraphs.

### Animals, blood sampling and processing

The study was carried out in compliance with the ARRIVE guidelines. Animals used in the study were kept in compliance with the Italian and European legislation for pig production (DLgs 122/2011 and Council Directive 2008/120/EC). Ethical review and approval were waived for this study because animals were not specifically raised or treated for the study, and blood samples were collected post-mortem during regular economic slaughter provided in a professional slaughterhouse, with procedures following guidelines established by Italian and European Union regulations for animal care and slaughter (Council Regulation (EC) No 1099/2009).

A total of 36 healthy pigs were included in this study: 12 Italian Landrace (ILA; five castrated males and seven gilts), 12 Italian Large White (ILW; six castrated males and six gilts) and 12 Italian Duroc (IDU; six castrated males and six gilts) pigs. All pigs were derived from the sib-testing programmes managed by the National Association of Pig Breeders (ANAS). Animals were provided food and water semi-*ad libitum* and housed under identical conditions in the same location until they reached approximately 155 ± 5 kg live weight. Animals were then transported to a commercial abattoir and slaughtered all on the same day in the morning at around 8:00 a.m. Blood was collected in EDTA tubes (Vacutest Kima, Padua, Italy) immediately after jugulation directly from the draining carotid, processed within two hours and stored at −80 °C for metabolomic analyses. Processing included inversion of the tubes eight to ten times and then centrifugation at 2,420 × g for 10 min at + 4 °C. Animals were raised and treated uniformly to ensure that observed metabolic differences reflected breed-specific genetic backgrounds.

## Metabolomic profiling of Porcine plasma samples

Targeted metabolomics, based on the Biocrates AbsoluteIDQ^™^ p180 kit (BIOCRATES Life Science AG, Innsbruck, Austria), was used to analyse porcine plasma samples. This Biocrates platform analyses 186 metabolites across seven analyte subclasses: 40 acylcarnitines (AC), 21 amino acids (AA), 19 biogenic amines (BA), 1 monosaccharide (namely hexoses, including glucose), 14 lyso-phosphatidylcholines (LysoPC), 76 phosphatidylcholines (PC; aa: diacyl; ae: acyl-alkyl) and 15 sphingomyelins (SM). All samples were processed on a single Biocrates plate alongside two replicated samples for quality control during quantification assessment. The analyses were conducted using a Serie 200 HPLC system (Perkin Elmer, Inc., Waltham, MA, USA) coupled with an API 4000 QTrap mass spectrometer (AB-Sciex, Framingham, MA, USA).

Following a similar approach to our previous work^10^, data filtering involved a three-step procedure to assess each metabolite: (i) intraplate coefficient of variation, (ii) percentage of missing values (NA) and zero values in the dataset, and (iii) identification of outlier samples. Subsequently, metabolite levels were regressed over covariates (fixed effects: sex and carcass weight) to eliminate any potential confounding effects. Briefly, the model was: $\:{y}_{i}=\:{\beta\:}_{0}\:+{\beta\:}_{w}{w}_{i}+{\beta\:}_{s}{s}_{i}+{\xi\:}_{i}\:$, where y_i_ denotes the metabolite concentration for the i^th^ animal, β_0_ denotes the intercept term, w_i_ indicates the weight of the i^th^ animal, s_i_ indicates the sex of the i^th^ animal, β_w_ and β_s_ are the corresponding regression coefficients and ξ_i_ is the error term. Residuals were then obtained and used in data analysis. Regression analysis was performed on the overall dataset (comprising all animals from the three breeds). Additional information on the targeted metabolites, analytical methods and statistical models can be found in^10^ and Supplementary Table S1. All analyses were conducted using R v.4.2.3^23^.

## Identification of differentially abundant metabolites

For each metabolite, relative concentration difference between breeds (Δ%^10,19^; was first calculated. Differential abundance was then assessed using supervised multivariate modelling with Boruta, a wrapper for a random forest classification algorithm^24^. Pairwise comparisons (pairwise setup; ILA vs. IDU, IDU vs. ILW, and ILA vs. ILW; hereafter ILA-IDU, IDU-ILW, and ILA-ILW) were performed, modelling the breed as the response variable and metabolite residuals as predictors. Metabolites classified as “Confirmed” were considered discriminant. Boruta was parameterized as in our previous studies^19,22^ and validated through (i) five runs with different random seeds and (ii) leave-one-out (LOO) cross-validation. In pairwise analyses (*N* = 24), LOO iteratively excluded one sample, and five Boruta runs were applied to the remaining *N*−1 samples, yielding 120 runs (5 × 24). The same procedure was extended to the full dataset (*N* = 36) in a multi-class setup, resulting in 180 runs (5 × 36).

The analysis was conducted in Python v3.11.7, using the BORUTA_py and scikit_learn packages. Based on the results of these latter validation steps, metabolites were retained and categorized into two subsets: (1) “highly reliable”, i.e. selected in all runs (120/120 in pairwise comparisons or 180/180 in the multi-class setup; 100%) and (2) “moderately reliable”, i.e. selected in at least 108/120 runs (≥ 90%) in pairwise setup or ≥ 144/180 in the multi-class setup. Metabolites that did not meet these criteria were labelled as unstable contributors to breed discrimination.

Following our previous studies^19,22^, selected metabolites were then used as input for a random forest analysis to obtain (i) the Mean Decrease Gini (MDG) value, indicative of the importance of each metabolite and (ii) the Out-Of-Bag (OOB) score and error, two indexes measuring the prediction performance of the random forest (and consequently Boruta). The random forest analysis was conducted in R v.4.2.3 (package randomForest^25^), with the option to grow 1,000 trees. Additional details are provided in our previous studies^19,22^.

Metabolites were further evaluated for their predictive performance using a ROC-AUC value obtained from the function *auc* of the R package pROC^26^.

Differences in metabolite levels between pairs of breeds were also tested in a univariate manner by applying the Mann-Whitney U Test (MWU).

In addition to the OOB values, the discriminant power of selected metabolites was evaluated by performing PCA on the datasets both before and after metabolite selections. For PCA, the data were scaled to have unit variance. The analyses run in R v.4.2.3^23^.

## Boruta-based re-analysis of the previously IDU-ILW investigated dataset

In our previous pilot study^10^, we profiled the metabolome of 12 IDU and 12 ILW pigs using both plasma and serum samples that were analysed with the Biocrates p180 kit. After filtering, the dataset contained 154 metabolites, which were originally analysed using sparse Partial Least Squares Discriminant Analysis (sPLS-DA) on residuals obtained from linear models that accounted for sex and carcass weight. Here, we re-analysed the same dataset using our Boruta-based pipeline, as described above, to validate the previously obtained results and to facilitate direct comparison with the new ILW and IDU independent dataset, analysed in the current study. Both plasma and serum samples were included in this re-analysis.

## Results

### General overview of the metabolomic panel

After filtering, the final dataset included a total of 181 out of 186 metabolites from the Biocrates panel. The five excluded metabolites had the following characteristics: (i) one metabolite (C12) had a coefficient of variation greater than 20 across quality controls (replicates), (ii) three metabolites (3-nitro-tyrosine, cis-4-hydroxyproline and phenylethylamine) had concentrations lower than the limit of detection (LOD) across quality controls (replicates) and (iii) one metabolite (aspartate) had concentrations lower than the LOD for more than 30% of the samples. Supplementary Table S1 lists evaluated metabolites, their coefficients of variation, average abundance, range of variation, and standard deviation in the three different breeds.

**Fig. 2:**
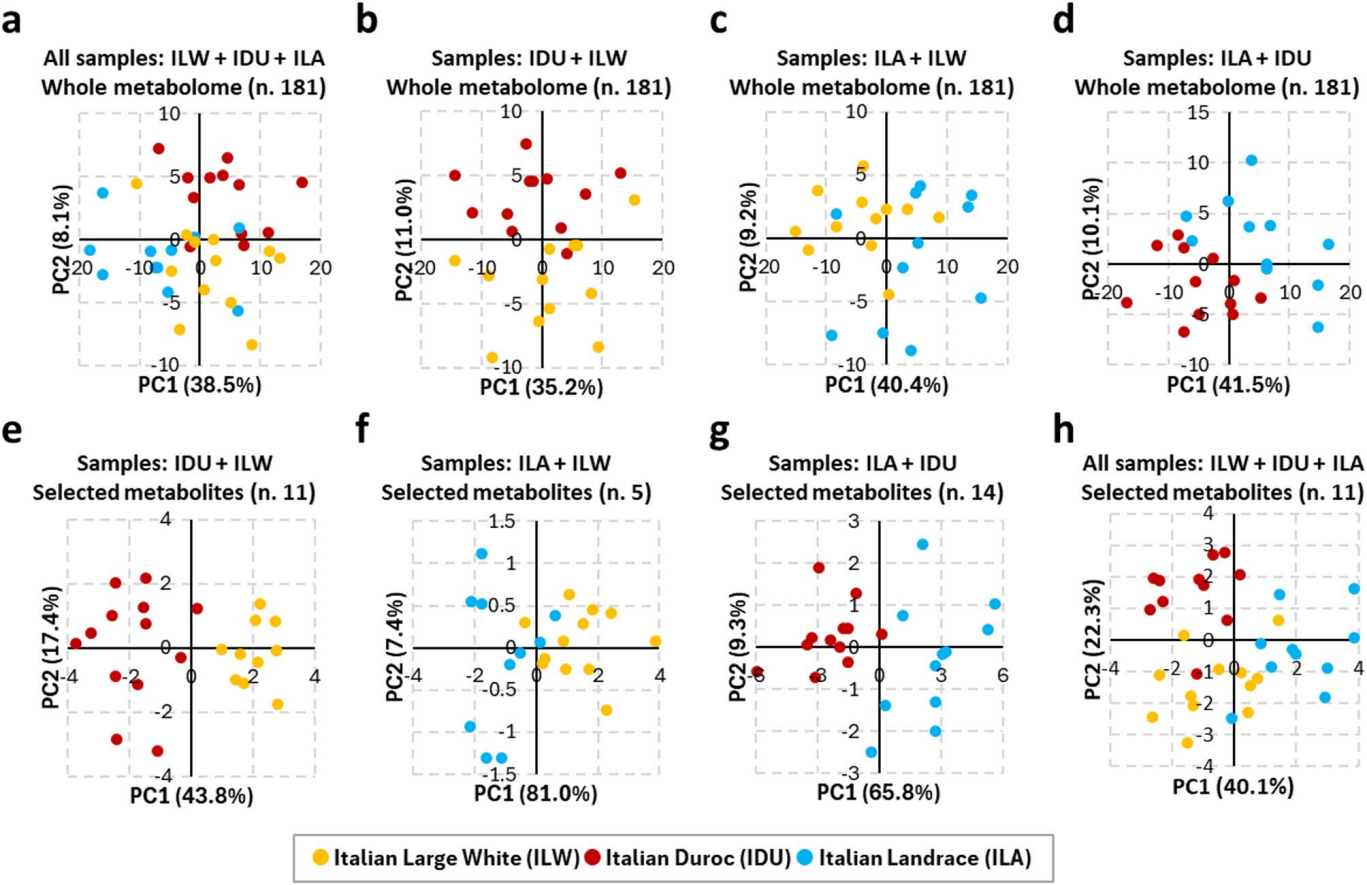
Results of Principal Component Analyses (shown for Principal Component 1 and Principal Component 2, PC1 and PC2) based on **(a-d)** the whole metabolomic profiles and **(e-h)** Boruta-selected metabolites.

## Metabolomic differences between Italian heavy pig breeds

Preliminary PCA based on the entire metabolomics profile (n. 181 metabolites) was conducted to explore differences between breeds. Analyses were performed on both the global dataset level (including all pigs from the three breeds; Fig. 2a) and on the datasets used in pairwise comparisons (each dataset containing pigs from two breeds only; Fig. 2b-d). Overall, while some clusters representing breeds are visible, there is a noticeable overlap, indicating that the whole metabolite profile contains several metabolic features shared across breeds. Additionally, despite the fact that the first principal component (PC1) captured most of the variance (ranging from 35.2 to 41.5%), it is not the primary axis separating the breeds (Fig. 2b-d).

**Fig. 3:**
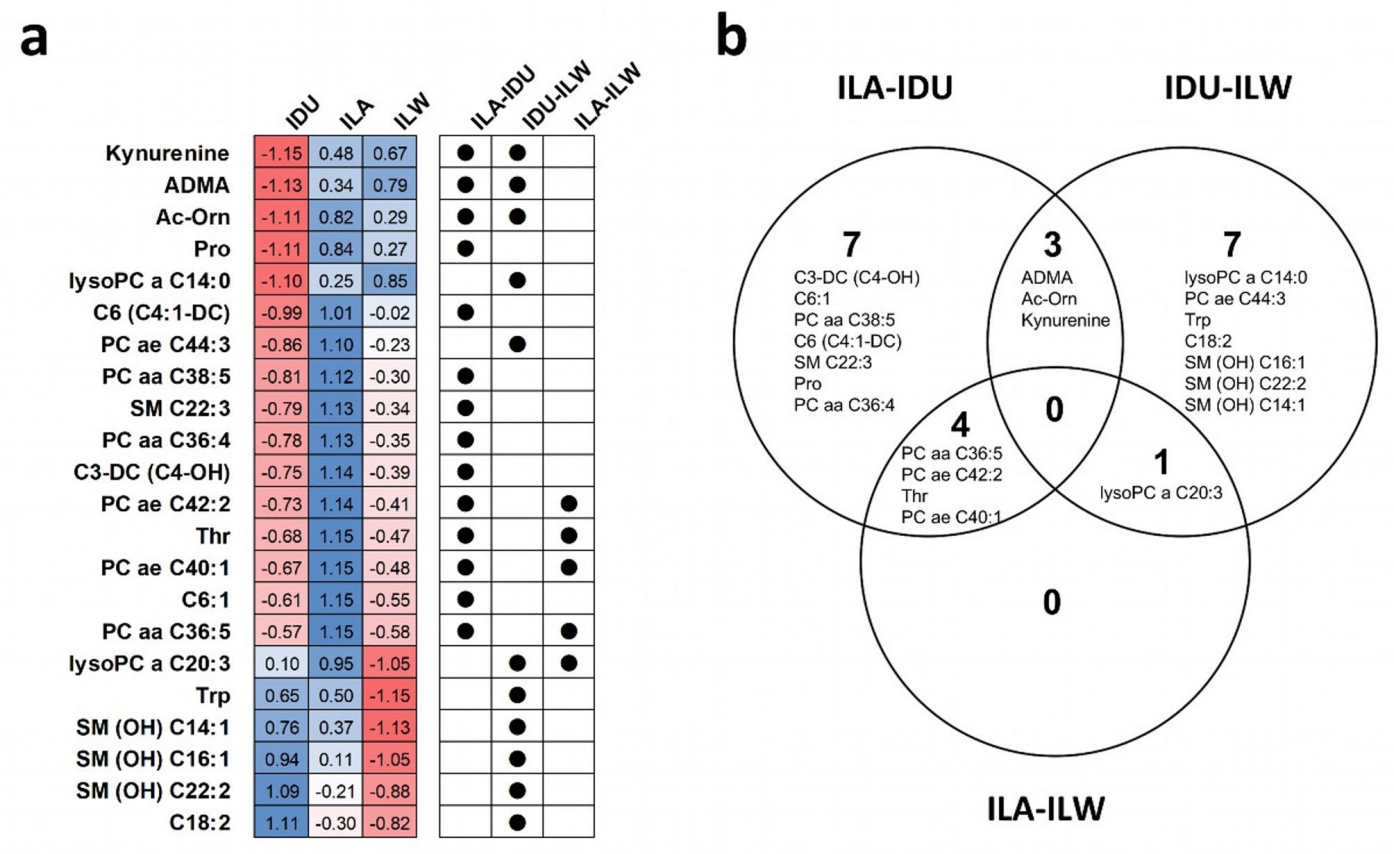
Differentially abundant metabolites (n. 22) identified by Boruta in the pairwise analyses of pig breeds (Italian Large White, ILW; Italian Landrace, ILA; Italian Duroc, IDU). **(a)** Heatmap of metabolites concentrations (residuals; scaled) and their source dataset (analysed dataset). **(b)** Venn diagram showing the overlap of metabolites among the analysed breed datasets. Full names of metabolites are provided in Supplementary Table S1.

Boruta was then used to identify breed-related metabolites. Analyses returned a total of 14, 11 and 5 metabolites selected in the ILA-IDU, IDU-ILW and ILA-ILW comparisons (Table 1), respectively. The proportion of runs in which each metabolite was retained is reported in Supplementary Table S2. Adoption of these metabolites for PCA (Fig. 2e-g) resulted in more pronounced separation among breeds due to PC1, explaining variance that increased up to 81%, and suggesting these metabolites as the among the drivers of the observed metabolic divergence among breeds. Overall, the total number of selected metabolites was 22, with some metabolites contributing as discriminant in two (or more) pairwise comparisons (Table 1; Fig. 3). Based on their chemical class, these 22 metabolites included 4 acylcarnitines, 3 amino acids, 3 biogenic amines, 2 lyso-phosphatidylcholines, 6 phosphatidylcholines and 4 sphingomyelins.

**Table 1:** Differentially abundant metabolites (n. 22) identified by Boruta in the analysed pig breeds (Italian large White, ILW; Italian Landrace, ILA; Italian Duroc, IDU). Metabolites selected in each pairwise comparison are reported sorted by the mean decrease Gini (MDG) value.

					Mann-Whitney U Test^6^		
Metabolite^1^	Analyte class^2^	Δ%^3^	MDG^4^	AUC^5^	ILA-IDU	IDU-ILW	ILA-ILW
**Comparison: ILA-IDU**							
PC aa C36:5 ^ILA−ILW, MC^	GP	−66	1.60	0.97	1.4E-04^*^	8.9E-01	7.4E-04^*^
PC ae C42:2 ^ILA−ILW, MC^	GP	−72	1.37	0.97	4.7E-05^*^	2.0E-01	2.1E-04^*^
Ac-Orn ^IDU−ILW, MC^	BA	−107	1.24	0.96	4.5E-05^*^	2.7E-04^*^	2.9E-01
PC aa C38:5	GP	−32	0.81	0.91	2.4E-04^*^	4.4E-01	7.9E-02
C6 (C4:1-DC) ^MC^	AC	−29	0.79	0.89	6.0E-04^*^	3.0E-02	6.0E-02
SM C22:3	SM	−55	0.70	0.86	6.3E-04^*^	4.8E-01	2.7E-02
Kynurenine ^IDU−ILW, MC^	BA	−105	0.64	0.86	6.7E-03^*^	1.5E-06^*^	6.5E-01
Thr ^ILA−ILW^	AA	−29	0.62	0.89	1.5E-03^*^	4.4E-01	1.3E-03^*^
PC ae C40:1 ^ILA−ILW, MC^	GP	−77	0.60	0.91	1.1E-04^*^	6.3E-01	9.8E-04^*^
C6:1	AC	−15	0.57	0.91	3.4E-04^*^	1.0E + 00	1.6E-02
Pro	AA	−31	0.55	0.89	2.2E-04^*^	1.0E-02	1.9E-01
PC aa C36:4	GP	−45	0.54	0.91	1.5E-04^*^	4.8E-01	3.2E-02
ADMA ^IDU−ILW, MC^	BA	−42	0.52	0.91	1.9E-04^*^	3.7E-04^*^	4.5E-01
C3-DC (C4-OH)	AC	−31	0.45	0.89	1.5E-04^*^	4.8E-01	1.1E-02
**Comparison: IDU-ILW**							
Kynurenine ^ILA−IDU, MC, A, B^	BA	56	3.00	0.99	2.8E-03^*^	1.5E-06^*^	6.5E-01
ADMA ^ILA−IDU, MC^	BA	32	1.22	0.90	4.0E-04^*^	3.7E-04^*^	4.5E-01
SM (OH) C14:1 ^MC, A^	SM	−21	1.08	0.86	5.7E-01	1.8E-03^*^	1.6E-02
Ac-Orn ^ILA−IDU, MC, A, B^	BA	45	0.95	0.91	2.8E-05^*^	2.7E-04^*^	2.9E-01
lysoPC a C20:3 ^ILA−ILW, MC^	GP	−28	0.86	0.85	9.1E-02	2.9E-03^*^	6.7E-05^*^
SM (OH) C22:2 ^MC^	SM	−22	0.86	0.82	2.7E-02	6.8E-03^*^	1.5E-01
SM (OH) C16:1 ^A, B^	SM	−21	0.75	0.85	3.5E-01	2.9E-03^*^	6.9E-02
lysoPC a C14:0	GP	10	0.72	0.85	2.7E-02	2.9E-03^*^	3.8E-01
Trp ^B^	AA	−24	0.71	0.88	9.3E-01	1.1E-03^*^	1.9E-02
PC ae C44:3	GP	13	0.70	0.76	1.3E-02	2.8E-02^*^	3.7E-02
C18:2 ^B^	AC	−54	0.60	0.88	4.4E-02	8.6E-04^*^	2.9E-01
**Comparison: ILA-ILW**							
lysoPC a C20:3 ^IDU−ILW, MC^	GP	−51	3.53	0.95	9.1E-02	2.9E-03^*^	6.7E-05^*^
PC ae C42:2 ^ILA−IDU^	GP	−56	2.11	0.92	1.8E-05^*^	2.0E-01	2.1E-04^*^
Thr ^ILA−IDU^	AA	−25	1.97	0.88	7.4E-04^*^	4.4E-01	1.3E-03^*^
PC ae C40:1 ^ILA−IDU, MC^	GP	−67	1.70	0.89	4.0E-04^*^	6.3E-01	9.8E-04^*^
PC aa C36:5 ^ILA−IDU, MC^	GP	−67	1.68	0.89	1.8E-05^*^	8.9E-01	7.4E-04^*^

^1^ Molecule selected by Boruta in the specified comparison. Full names are provided in Supplementary Table S1. If Boruta selected a molecule in other breed comparisons, the information is presented as a superscript in the format ILA-IDU, IDU-ILW and ILA-ILW. Metabolites identified by using the multi-class approach are labelled with the superscript in the format “MC”. For the IDU-ILW comparison we report also if the metabolite was selected in the Boruta-based re-analysis of the 2016 IDU-ILW dataset^10^ (superscript in the format “A”) and/or in our previous study using an untargeted platform in a larger sample size^19^ (superscript in the format “B”).

^2^ Biological class of the molecule, as provided by Biocrates (AA: amino acid, AC: acylcarnitine, BA: biogenic amine, GP: glycero-phospholipid, SM: sphingomyelin).

^3^ Relative difference in concentration between pairs of breeds. For the ILA-IDU, IDU-ILW and ILA-ILW, a negative value indicates higher metabolite levels in IDU, ILW and ILW, respectively.

^4^ Mean Decrease Gini (MDG) value obtained from the random forest analysis.

^5^ Area Under the Curve (AUC) value obtained from the Receiver Operating Characteristic curve analysis.

^6^ *P*-values obtained at the Mann-Whitney U Test. The star symbol (*) is used to mark *p*-values < 0.01.

**Fig. 4:**
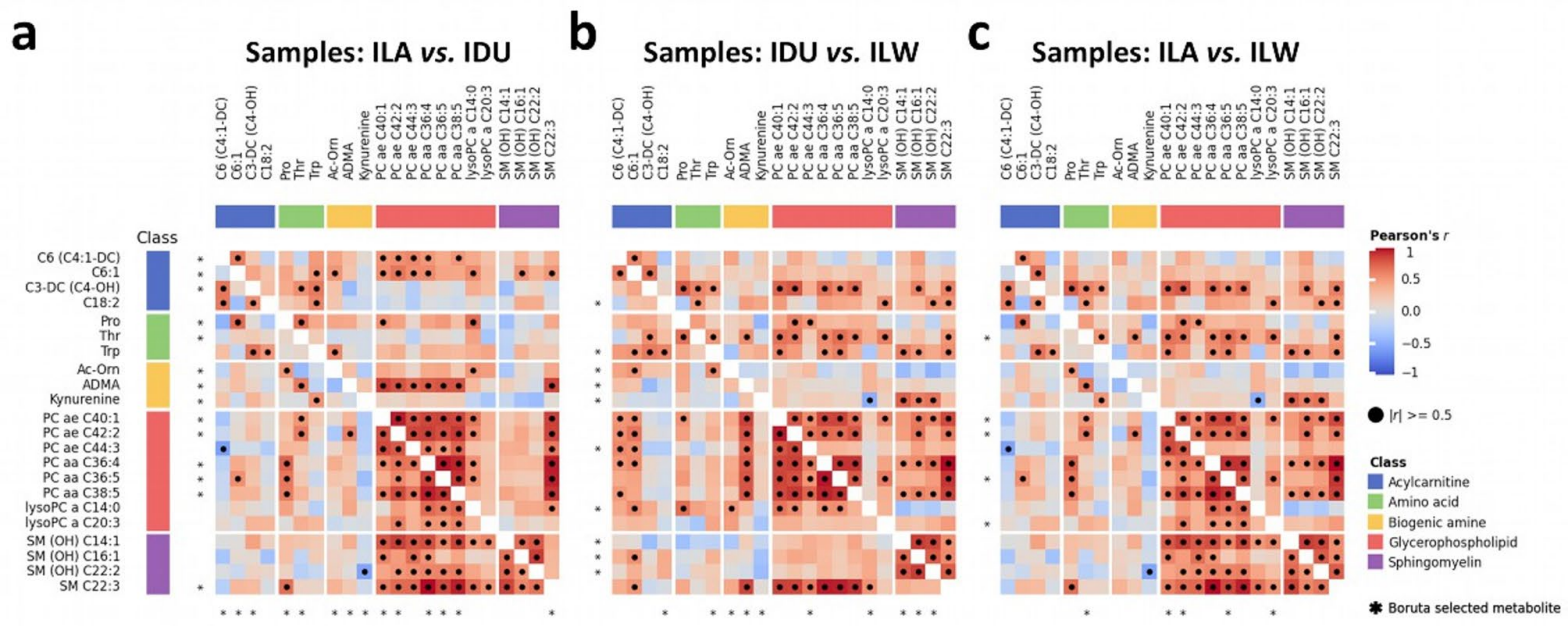
Pearson correlation matrices showing the within-breed relationships among the 22 metabolites identified by Boruta (pairwise comparisons) in the analysed pig breeds (Italian Large White, ILW; Italian Landrace, ILA; Italian Duroc, IDU). **(a)** ILA-IDU comparison; upper triangle: relationships in IDU pigs; lower triangle: relationships in ILA pigs. **(b)** IDU-ILW comparison; upper triangle: relationships in ILW pigs, lower triangle: relationships in IDU pigs. **(c)** ILA-ILW comparison; upper triangle: relationship in ILW pigs; lower triangle: relationships in ILA pigs. Metabolites selected in each comparison are indicated with the star (*) symbol. Relevant correlations (|*r*|≥0.5; regardless of their *p*-value) are indicated with the dot (●) symbol. Correlation values and associated *p*-values are reported in Supplementary Tables S3–S5.

In the ILA-IDU comparison, the 14 selected metabolites (Table 1) included all chemical classes mentioned above except for lysoPC. Within-breed relationships between metabolite abundances are given in Fig. 4a. Corresponding correlation coefficients and *p*-values are reported separately for ILA (Supplementary Table S3) and IDU (Supplementary Table S4) pigs. These metabolites remained stable throughout the discrimination process, passing all the LOO runs and forming a “highly reliable” metabolite set, except for three metabolites [C3-DC (C4-OH), C6:1 and ADMA] that were placed in the “moderately reliable” metabolite set (≥ 90% of LOO runs). In the quantitative evaluation of metabolite levels (Fig. 3a), it is noteworthy that all selected metabolites showed negative Δ% values, indicating lower metabolite levels in IDU compared to ILA pigs. Extreme values of Δ% (|Δ|% = 105) were observed for kynurenine and N-acetylornithine (Ac-Orn), two biogenic amines out of the three representing this metabolite class in the selected subset. All other metabolites had |Δ|% in the range of 15–77. In terms of discriminative power, when evaluated using AUC statistics, the values were high, ranging from 0.86 to 0.97, with the highest values (> 0.95) for Ac-Orn and two phosphatidylcholines (PC aa C36:5 and PC ae C42:2). These three metabolites also had the highest MDG values. The OOB error rate was 13% (misclassified pigs: two ILA and one ILW pigs). According to the Mann-Whitney U test, all metabolites showed *p*-values < 0.01, with nine remaining significant after correction for multiple testing (Bonferroni, 0.05/181). Three and four metabolites from this selection set were also identified in the IDU-ILW and ILA-ILW comparisons, respectively (Table 1; Fig. 3).

When comparing the IDU-ILW profiles, the 11 selected metabolites (Table 1) included all the chemical classes mentioned. Within-breed relationships between metabolite abundances are given in Fig. 4b. Corresponding correlation coefficients and *p*-values are reported separately for IDU (Supplementary Table S4) and ILW (Supplementary Table S5) pigs. The process of selecting metabolites for this dataset was less stable compared to the ILA-IDU comparison, with half of the selected metabolites belonging to the “highly reliable” set and the other half to the “moderately reliable” set. Quantitatively, half of the metabolite set had low levels in IDU pigs (positive Δ%), while the other half had high levels in IDU pigs (negative Δ%), with Δ% values ranging from + 56 to −54. Acylcarnitine C18:2 (higher levels in IDU pigs) and kynurenine (lower levels in IDU) represented the extreme values. The discriminative power of each selected metabolite, based on AUC values, was quite high, ranging from 0.76 to 0.99. High AUC values (≥ 0.90) were assigned to all three biogenic amines in this metabolite set: ADMA (AUC = 0.90), Ac-Orn (AUC = 0.91) and kynurenine (AUC = 0.99). The OOB error was very low, at 4% (misclassified pig: one ILW). It is worth noting that these three metabolites (ADMA, Ac-Orn and kynurenine) were also selected in the previous comparison (ILA-IDU) (Table 1; Fig. 3), showing lower values in IDU compared to ILW and ILA (and similar levels between ILA and ILW). According to the Mann-Whitney U test, all metabolites, except PC ae C44:3, showed *p*-values < 0.01, but only two (Ac-Orn and kynurenine) remained significant after correction for multiple testing.

In the ILA-ILW comparison, five metabolites were selected (Table 1): one amino acid (threonine), three phosphatidylcholines (PC aa C36:5, PC ae C40:1, and PC ae C42:2), and one lysophosphatidylcholine (lysoPC a C20:3). Within-breed relationships between metabolite abundances are given in Fig. 4c. Corresponding correlation coefficients and *p*-values are reported separately for ILA (Supplementary Table S3) and ILW (Supplementary Table S5) pigs. The small number of discriminative metabolites emphasizes the high similarity between the two breeds, as also seen in genetic analyses. However, it is important to note that only a portion of the plasma metabolome has been examined here, and this observation is specific to that context. This may explain the high error rate obtained in the random forest implementation: OOB was 18%, with one ILA and two ILW pigs misclassified. Quantitatively, all these metabolites had lower levels in the ILW pigs, with Δ% values ranging from − 25% to −67%. Interestingly, all five of these metabolites were also found to be discriminant features in previous pairwise comparisons (Table 1; Fig. 3): four (threonine and the phosphatidylcholines) and one (lysoPC a C20:3) in the ILA-IDU and IDU-ILW comparisons, respectively. The AUC values ranged from 0.88 to 0.95. The molecule lysoPC a C20:3 had the highest AUC and MDG values. According to the Mann-Whitney U test, all five metabolites showed *p*-values < 0.01, but only two (lysoPC a C20:3 and PC ae C42:2) remained significant after correction for multiple testing.

When all three breeds were analysed together using Boruta (multi class setup), 11 metabolites were identified (Table 1, Supplementary Table S2). These included: 2 acylcarnitines [C6 (C4:1-DC) and C6:1], 3 biogenic amines (Ac-Orn, kynurenine, and ADMA), 4 phosphatidylcholines (PC aa C36:5, PC ae C40:1, and PC ae C42:2), 1 lysophosphatidylcholine (lysoPC a C20:3), and 2 sphingomyelins [SM (OH) C22:2 and SM (OH) C14:1]. All these metabolites had also been identified in at least one of the pairwise breed comparisons (Table 1). A PCA plot based on this metabolite set is shown in Fig. 2h. Using this set, OOB error was approximately 20%. Notably, comparison with the pairwise analyses revealed that half of these discriminant metabolites (11 out of 22) were not retained in this multi-class analysis. These included: Trp (ILW-IDU), Thr (ILA-IDU and ILA-ILW), Pro (ILA-IDU), C3-DC (C4-OH) (ILA-IDU), C18:2 (ILW-IDU), PC aa C36:4 (ILA-IDU), PC aa C38:5 (ILA-IDU), PC ae C44:3 (IDU-ILW), lysoPC a C14:0 (IDU-ILW), SM (OH) C16:1 (IDU-ILW), and SM C22:3 (ILA-IDU).

## Boruta-based re-analysis of an independent IDU-ILW dataset

The previously investigated and independent IDU-ILW dataset^10^ from that analysed in the current study included 24 animals (12 ILW and 12 IDU pigs) with both plasma and serum metabolomic profiles, originally analysed using sPLS-DA. We re-analysed this dataset to evaluate the performance of our newly developed Boruta-based approach. In plasma, sPLS-DA identified three metabolites [Ac-Orn, kynurenine, SM (OH) C14:1], all confirmed by Boruta, which additionally detected SM (OH) C16:1, consistent with results obtained in the current and new dataset. Overall, these four metabolites were shared between the re-analysed and the new dataset. In serum, sPLS-DA identified four metabolites [Ac-Orn, SM (OH) C14:1, SM (OH) C16:1, SM C16:0], all confirmed by Boruta, with no additional metabolites detected. The proportion of runs in which each metabolite was retained is reported in Supplementary Table S6. PCA plots before and after metabolite selection are provided for plasma (Supplementary Fig. S1a-b) and serum (Supplementary Fig. S1c-d). For the random forest implementation, MDG values are given in Supplementary Table S6. The out-of-bag (OOB) error was low in plasma (12.5%) but higher in serum (30%).

## Discussion

In this study, we focused on the identification of metabolites that differ in concentration levels and can discriminate breeds when used in a classification task. Their identification is inherently connected to the underlying metabolic differences between breeds, reflecting the biological interpretation of abundance variation. The metabolites that were identified support and expand our understanding of the metabolome of heavy pigs. Specifically, the results pertain to the plasma metabolome of animals at approximately nine months of age, with a live weight of around 160 kg, which corresponds to the very late stage of the Italian heavy pig production system: the finishing and slaughtering phase, which is however quite similar in terminal pigs of other production systems. Blood samples (from which plasma was separated and used for metabolomic analysis) were collected at the abattoir just after the animals were slaughtered. Pigs at this stage meet the required carcass characteristics defined in the regulations for Protected Designation of Origin (PDO) dry-cured ham production^21^.

Metabolomics studies comparing Italian pig breeds have been limited so far, with targeted (Biocrates panel) and untargeted (Metabolon platform) approaches only used to compare ILW and IDU pigs^10,19^. By including ILA pigs representing a third genetic background, we extended our knowledge through a comparative analysis of metabolic profiles across heavy pig populations, providing a deeper understanding of breed-related molecular phenotypes. Although this was a pilot study with a limited sample size (12 pigs per breed), we designed the experiment to minimize both environmental and biological confounding factors, by considering: (i) standardized animal conditions, as all pigs were raised, fed and treated in the same way, and slaughtered on the same abattoir within 1 h (each animals were slaughtered within few minutes), (ii) a balanced sex ratio, to ensure sex-related metabolic differences were accounted for in the analysis and (iii) consistent analytical procedures, as samples were processed and stored in the same way by the same operator, and metabolomic profiling was conducted on a single Biocrates plate, with the analysis running continuously for 12 h. Furthermore, since previous studies have already compared IDU with ILW pig populations^10,19^, we can also consider part of this study as a replication study for these two breeds.

To identify differentially abundant metabolites, we utilised a statistical pipeline based on Boruta, a random forest wrapper. This approach was chosen because Boruta addresses the *all-relevant feature selection problem*^24^, allowing the identification of the complete set of informative metabolites for prediction, rather than only the minimal set required for a predictive model (*minimal-optimal model*)^24^, as in sparse Partial Least Squares Discriminant Analysis (sPLS-DA) used in our previous study^10^. Our strategy was to test breed differences in a pairwise manner, so that any metabolite showing a difference between two groups could be considered informative and selected. In contrast, the multi-class setup (all three breeds analysed together) requires simultaneous separation of classes, and thus only metabolites that consistently contribute to distinguishing all groups are retained. As a result, fewer metabolites were selected in the three-class case, since the selection criteria are stricter. Overall, the pairwise analyses identified 22 discriminant metabolites, of which 11 were also retained by the multi-class setup. Notably, 10 of the 11 metabolites not retained were detected in only one specific pairwise comparison, reflecting their ability to separate one breed from another but not all three simultaneously.

Consistent with our previous pilot study using the same Biocrates panel^10^, most of the metabolites identified by Boruta as differentiating IDU and ILW pigs (IDU-ILW comparison) were confirmed. Specifically, Ac-Orn, kynurenine, SM (OH) C14:1 and SM (OH) C16:1^10^ were identified, reinforcing our understanding of metabolic differences between these two breeds. The relevance of these four metabolites was further confirmed by re-analysis of our previous IDU-ILW dataset^10^ using the novel Boruta-based pipeline, supporting the robustness of both the analytical platform and the data analysis approach. Ac-Orn, kynurenine and SM (OH) C16:1 were also confirmed using the untargeted platform that we recently utilised to compare the plasma metabolome of the same two pig breeds^19^, along with tryptophan (trp) and linoleoylcarnitine (C18:2). Notably, tryptophan and kynurenine, two molecules of the kynurenine pathway (involved in tryptophan metabolism), emerged as key differentiating metabolites. These molecules play important roles in brain functions, immunity, and reproduction^27^. The difference in kynurenine levels between these two breeds can be attributed to their distinct genetic backgrounds, as our recent metabolite genome-wide association studies (mGWAS) identified a major metabolite quantitative trait locus (mQTL) for kynurenine in both breeds^20^. This mQTL includes the kynurenine 3-monooxygenase (*KMO*) gene, that encodes a key enzyme responsible for converting L-kynurenine to 3-hydroxy-L-kynurenine. Specifically, two major *KMO* haplotypes with opposite frequencies were found in ILW and IDU pigs, explaining the variations in kynurenine levels^20^. Similarly, genomic associations for Ac-Orn, ADMA and C18:2 were also identified in one or both breeds^20^. No associations were found in mGWAS for the other metabolites. However, it is possible that the lack of associations reported in the mGWAS study is due to the small sample size of the Duroc population analysed, which may have resulted in an underpowered experiment. The IDU-ILW comparison also revealed several sphingomyelins, which are molecules involved in cell signaling, membrane integrity, stability, and energy storage. This finding could be related to the intermuscular fat deposition process that characterises the Duroc breed^28^.

Similarly to the IDU-ILW comparison, which was characterized by 11 differentially abundant metabolites, the ILA-IDU comparison revealed even more differences (14 metabolites). In contrast, the ILA-ILW comparison showed only a few differences (5 metabolites), which was expected given the genetic closeness and selection histories of these two breeds^28,29,30^: Duroc pigs are generally selected to improve intramuscular fat (IMF), meat quality and robustness, while Large White and Landrace pigs are selected for lean growth, reproductive performance, and efficiency^28,31^. The IDU-ILW and ILA-IDU comparisons shared metabolites, including Ac-Orn, kynurenine and ADMA. The levels of these metabolites were generally lower in IDU and higher in ILW and ILA pigs, suggesting their potential as Duroc-specific biomarkers. Moreover, similar *KMO* allele frequencies observed in ILW and ILA pigs^20^ might explain the kynurenine differences seen specifically in the IDU-ILW comparison. Except for Ac-Orn and kynurenine, for most of the other differentially abundant metabolites no mQTL were detected, possibly due to the limited sample size of the Duroc breed^20^. Notably, the ILA-IDU comparison presented several differentially abundant acylcarnitines (AC) [i.e. C6 (C4:1-DC), C6:1, and C3-DC (C4-OH)], which were found at lower levels in IDU (and ILW) compared to ILA pigs. Acylcarnitines are metabolites synthesized within fatty acid metabolism (β-oxidation) and serve as markers of energy metabolism, reflecting lipid mobilization and utilization^32^. Notably, the identified molecular differences align with known phenotypic characteristics of the respective breeds. Italian Duroc pigs have higher intramuscular fat (IMF) and intermuscular fat content, meaning the muscle tissue has an internal energy reserve in the form of fatty acids. On the contrary, being characterized by less IMF, ILW and ILA pigs need to break down fat stored in adipose tissue via lipolysis (energy reserve), release free fatty acids into the blood, and transport them to muscles to support the energy demand^33^. Thus, when muscles rely on external fatty acids, fatty acid oxidation increases, producing and increasing acylcarnitines levels. In addition, a high level of these molecules not only suggest an enhanced lipid metabolism but may also indicate incomplete fatty acid β-oxidation and the subsequent acylcarnitine accumulation^34,35^. Italian Large White pigs, despite having low IMF content, showed acylcarnitine levels comparable to IDU pigs, possibly due to a more efficient balance between fat mobilization and oxidation, limiting acylcarnitine accumulation in the blood. Italian Landrace pigs were also characterised by elevated levels of several phosphatidylcholines (PC), essential components of cell membranes and relevant for lipid transportation and cell signalling processes. In particular, they are key components of lipoproteins (especially Very Low Density Lipoprotein), which transport lipids from the liver to tissues. Thus, the concurrent rise in both PC and AC in ILA pigs supports the hypothesis of enhanced lipid mobilization, in contrast to IDU and ILW pigs, where lipid oxidation may occur more directly within tissues. Notably, all phosphatidylcholines differentiating ILW from ILA pigs were also involved in the IDU-ILA comparison and not in the IDU-ILW, reinforcing the ILA-specific lipid profile. However, lysoPC a C20:3 had an unusual pattern, showing similar levels in IDU and ILA, despite most lysoPCs following the broader PC trends in these two breeds. It is noteworthy that a major ILW-specific mQTL appeared to regulate this metabolite, as well as PC ae C42:2 and PC ae C40:1, while PC aa C36:5 was linked to an mQTL unique to the IDU breed^20^. In order to better support these findings and fully explore the gene-metabolite relationships, it would be valuable to include gene expression data as an additional layer of intermediate molecules. This would allow for a better understanding of the regulatory networks and gene expression changes that drive these metabolic processes.

In summary, breed-related metabolic profiles emerged, aligning with known characteristics of the breeds (such as robustness and fat deposition potential). Specifically, IDU pigs are marked by diminished biogenic amine levels, ILW pigs by lower sphingomyelin levels, and ILA by elevated acylcarnitine and phosphatidylcholines levels. These findings suggest different breed-related energy and lipid metabolism strategies.

## Conclusions

This study is the first to provide an integrated metabolomic characterisation of three Italian pig breeds: Italian Duroc, Italian Landrace, and Italian Large White. It offers a comprehensive overview of their metabolism and shed light on breed-related metabolic signatures, derived from their different genetic backgrounds. Although this study analyses a relatively limited number of metabolites (approximately 180) in one biofluid (the plasma), the results show potential metabolic biomarkers that reflect important molecular characteristics, unique to each breed. These findings could be used to develop new molecular phenotypes useful in pig breeding and selection and to explore breed-precision feeding strategies. Other studies are necessary to further characterise the metabolome profiles of pig breeds and apply them to practical solutions that improve sustainability in pig farming.

## Supplementary Information

Below is the link to the electronic supplementary material.

Supplementary Material 1

## Data Availability

The data that support the study findings are publicly available at: (10.5281/zenodo.15301582).
