# Supplementary material for: Comparative analysis of targeted metabolomic profiles reveals plasma metabolite differences across three Italian heavy pig breeds
Source: Sci Rep. 2025 Nov 10;15:39296. doi: 10.1038/s41598-025-23058-z (PMC12603176; doi:10.1038/s41598-025-23058-z)
Supplement: Supplementary file 1 — Supplementary Material 1 [file 41598_2025_23058_MOESM1_ESM.docx]

**Supplementary Information for:**

**Comparative analysis of targeted metabolomic profiles reveals plasma metabolite differences across three Italian heavy pig breeds**

Samuele Bovo^1^, Matteo Bolner^1^, Giuseppina Schiavo^1^, Flaminia Fanelli^2^, Giuliano Galimberti^3^, Francesca Bertolini^1^, Anisa Ribani^1^, Stefania Dall’Olio^1^, Paolo Zambonelli^1^, Uberto Pagotto^2^, Luca Fontanesi^1*^

^1^ Animal and Food Genomics Group, Division of Animal Sciences, Department of Agricultural and Food Sciences, University of Bologna, Viale Giuseppe Fanin 46, 40127 Bologna, Italy

^2^ Department of Surgical and Medical Sciences, Endocrinology Unit, University of Bologna, Via Giuseppe Massarenti 9, 40138 Bologna, Italy

^3^ Department of Statistical Sciences "Paolo Fortunati", University of Bologna, Via delle Belle Arti 41, 40126 Bologna, Italy

* Corresponding author

E-mail: [luca.fontanesi@unibo.it](mailto:luca.fontanesi@unibo.it) (LF)

**Figure S1**. Results of Principal Component Analyses (shown for Principal Component 1 and Principal Component 2; PC1 and PC2) based on (a-b) the whole metabolomic profiles and (c-d) Boruta-selected metabolites. Dataset originally described in Bovo et al. [10].


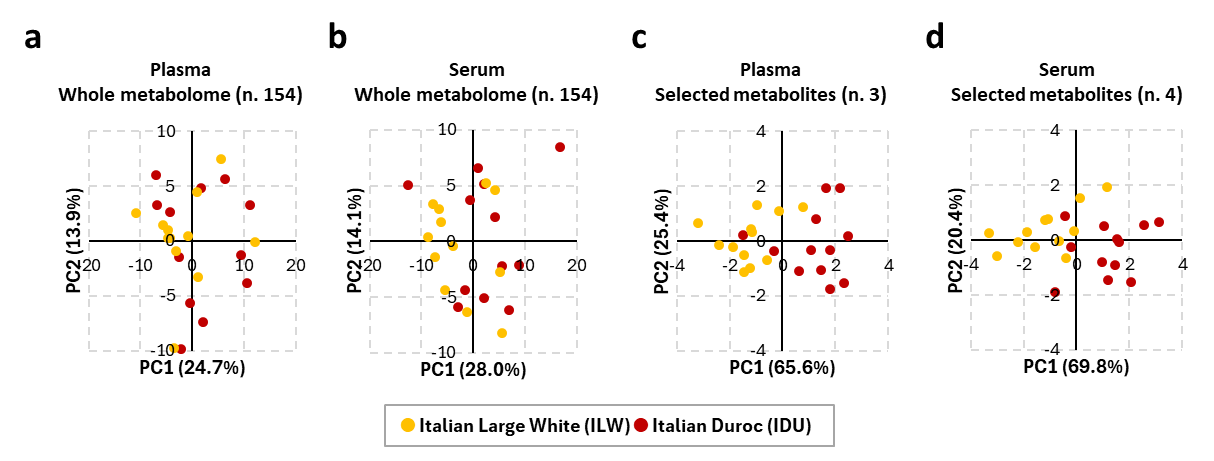


**Table S1**. Metabolites included in the study. Summary statistics of metabolite levels for the Italian Duroc, Italian Landrace and Italian Large White populations.

|  |  |  |  |  | **Italian Duroc** | | **Italian Landrace** | | **Italian Large White** | |
| --- | --- | --- | --- | --- | --- | --- | --- | --- | --- | --- |
| **Metabolite** | **Biochemical name** | **Metabolite class** | **Coefficient of variation** | **Quality control** | **Mean** | **s.d.** | **Mean** | **s.d.** | **Mean** | **s.d.** |
| C0 | Carnitine | Acylcarnitine | 8.65 | RETAINED | 6.902 | 1.107 | 6.489 | 1.064 | 6.073 | 1.09 |
| C10 | Decanoylcarnitine | Acylcarnitine | 4.46 | RETAINED | 0.085 | 0.011 | 0.084 | 0.009 | 0.079 | 0.009 |
| C10:1 | Decenoylcarnitine | Acylcarnitine | 6.62 | RETAINED | 0.042 | 0.005 | 0.046 | 0.006 | 0.043 | 0.006 |
| C10:2 | Decadienylcarnitine | Acylcarnitine | 4.62 | RETAINED | 0.026 | 0.004 | 0.028 | 0.004 | 0.032 | 0.008 |
| C12 | Dodecanoylcarnitine | Acylcarnitine | 43.51 | DISCARDED | 0.215 | 0.202 | 0.422 | 0.199 | 0.73 | 0.54 |
| C12-DC | Dodecanedioylcarnitine | Acylcarnitine | 6.01 | RETAINED | 0.213 | 0.014 | 0.221 | 0.012 | 0.213 | 0.012 |
| C12:1 | Dodecenoylcarnitine | Acylcarnitine | 2.48 | RETAINED | 0.038 | 0.007 | 0.046 | 0.007 | 0.054 | 0.016 |
| C14 | Tetradecanoylcarnitine | Acylcarnitine | 14.29 | RETAINED | 0.018 | 0.004 | 0.018 | 0.004 | 0.016 | 0.003 |
| C14:1 | Tetradecenoylcarnitine | Acylcarnitine | 5.33 | RETAINED | 0.024 | 0.006 | 0.023 | 0.008 | 0.021 | 0.006 |
| C14:1-OH | Hydroxytetradecenoylcarnitine | Acylcarnitine | 3.23 | RETAINED | 0.007 | 0.001 | 0.008 | 0.001 | 0.009 | 0.001 |
| C14:2 | Tetradecadienylcarnitine | Acylcarnitine | 10.45 | RETAINED | 0.007 | 0.002 | 0.007 | 0.002 | 0.008 | 0.002 |
| C14:2-OH | Hydroxytetradecadienylcarnitine | Acylcarnitine | 9.09 | RETAINED | 0.008 | 0.001 | 0.008 | 0.001 | 0.009 | 0.002 |
| C16 | Hexadecanoylcarnitine | Acylcarnitine | 7.23 | RETAINED | 0.029 | 0.009 | 0.027 | 0.007 | 0.027 | 0.006 |
| C16-OH | Hydroxyhexadecanoylcarnitine | Acylcarnitine | 14.29 | RETAINED | 0.011 | 0.002 | 0.012 | 0.002 | 0.011 | 0.002 |
| C16:1 | Hexadecenoylcarnitine | Acylcarnitine | 3.8 | RETAINED | 0.027 | 0.006 | 0.027 | 0.006 | 0.033 | 0.01 |
| C16:1-OH | Hydroxyhexadecenoylcarnitine | Acylcarnitine | 5.26 | RETAINED | 0.006 | 0.002 | 0.006 | 0.001 | 0.006 | 0.001 |
| C16:2 | Hexadecadienylcarnitine | Acylcarnitine | 13.04 | RETAINED | 0.006 | 0.001 | 0.006 | 0.001 | 0.006 | 0.001 |
| C16:2-OH | Hydroxyhexadecadienylcarnitine | Acylcarnitine | 13.04 | RETAINED | 0.008 | 0.001 | 0.008 | 0.001 | 0.008 | 0.001 |
| C18 | Octadecanoylcarnitine | Acylcarnitine | 4.92 | RETAINED | 0.024 | 0.009 | 0.02 | 0.007 | 0.021 | 0.004 |
| C18:1 | Octadecenoylcarnitine | Acylcarnitine | 7.55 | RETAINED | 0.033 | 0.012 | 0.03 | 0.015 | 0.027 | 0.01 |
| C18:1-OH | Hydroxyoctadecenoylcarnitine | Acylcarnitine | 11.76 | RETAINED | 0.018 | 0.002 | 0.016 | 0.002 | 0.016 | 0.002 |
| C18:2 | Octadecadienylcarnitine | Acylcarnitine | 0 | RETAINED | 0.016 | 0.005 | 0.011 | 0.003 | 0.011 | 0.003 |
| C2 | Acetylcarnitine | Acylcarnitine | 4.97 | RETAINED | 1.353 | 0.269 | 1.47 | 0.26 | 1.309 | 0.577 |
| C3 | Propionylcarnitine | Acylcarnitine | 6.48 | RETAINED | 0.09 | 0.014 | 0.086 | 0.018 | 0.08 | 0.026 |
| C3-DC (C4-OH) | Hydroxybutyrylcarnitine | Acylcarnitine | 7.87 | RETAINED | 0.04 | 0.007 | 0.052 | 0.01 | 0.042 | 0.008 |
| C3-OH | Hydroxypropionylcarnitine | Acylcarnitine | 7 | RETAINED | 0.092 | 0.008 | 0.095 | 0.011 | 0.087 | 0.009 |
| C3:1 | Propenonylcarnitine | Acylcarnitine | 4 | RETAINED | 0.012 | 0.002 | 0.012 | 0.001 | 0.012 | 0.001 |
| C4 | Butyrylcarnitine | Acylcarnitine | 4.76 | RETAINED | 0.068 | 0.008 | 0.072 | 0.011 | 0.062 | 0.011 |
| C4:1 | Butenylcarnitine | Acylcarnitine | 8 | RETAINED | 0.03 | 0.003 | 0.029 | 0.002 | 0.028 | 0.003 |
| C5 | Valerylcarnitine | Acylcarnitine | 13 | RETAINED | 0.03 | 0.004 | 0.029 | 0.004 | 0.028 | 0.003 |
| C5-DC (C6-OH) | Glutarylcarnitine | Acylcarnitine | 0 | RETAINED | 0.017 | 0.003 | 0.023 | 0.003 | 0.022 | 0.005 |
| C5-M-DC | Methylglutarylcarnitine | Acylcarnitine | 8.93 | RETAINED | 0.054 | 0.005 | 0.053 | 0.005 | 0.054 | 0.004 |
| C5-OH (C3-DC-M) | Hydroxyvalerylcarnitine (Methylmalonylcarnitine) | Acylcarnitine | 5.41 | RETAINED | 0.102 | 0.007 | 0.105 | 0.007 | 0.107 | 0.008 |
| C5:1 | Tiglylcarnitine | Acylcarnitine | 14.29 | RETAINED | 0.027 | 0.004 | 0.03 | 0.004 | 0.029 | 0.002 |
| C5:1-DC | Glutaconylcarnitine | Acylcarnitine | 0 | RETAINED | 0.01 | 0.001 | 0.012 | 0.002 | 0.01 | 0.001 |
| C6 (C4:1-DC) | Hexanoylcarnitine | Acylcarnitine | 1.3 | RETAINED | 0.04 | 0.005 | 0.052 | 0.007 | 0.045 | 0.005 |
| C6:1 | Hexenoylcarnitine | Acylcarnitine | 7.69 | RETAINED | 0.022 | 0.002 | 0.026 | 0.002 | 0.022 | 0.003 |
| C7-DC | Pimelylcarnitine | Acylcarnitine | 5.75 | RETAINED | 0.013 | 0.002 | 0.015 | 0.004 | 0.013 | 0.002 |
| C8 | Octanoylcarnitine | Acylcarnitine | 0.9 | RETAINED | 0.067 | 0.007 | 0.069 | 0.005 | 0.068 | 0.006 |
| C9 | Nonaylcarnitine | Acylcarnitine | 12.5 | RETAINED | 0.029 | 0.005 | 0.032 | 0.003 | 0.029 | 0.005 |
| Ala | Alanine | Amino acid | 7.77 | RETAINED | 398 | 69.081 | 498.636 | 124.803 | 426.333 | 89.376 |
| Arg | Arginine | Amino acid | 1.44 | RETAINED | 99.308 | 16.696 | 126.909 | 14.563 | 112.917 | 15.795 |
| Asn | Asparagine | Amino acid | 16.17 | RETAINED | 42.058 | 6.677 | 49 | 8.385 | 48.708 | 10.064 |
| Asp | Aspartate | Amino acid | 7.23 | DISCARDED | 15.233 | 3.282 | 14.3 | 0.8 | 15.3 | 1.5 |
| Cit | Citrulline | Amino acid | 9.21 | RETAINED | 76.458 | 14.099 | 63.345 | 13.762 | 66.367 | 12.817 |
| Gln | Glutamine | Amino acid | 4.35 | RETAINED | 387.417 | 70.423 | 441.091 | 80.456 | 456.167 | 95.146 |
| Glu | Glutamate | Amino acid | 4.96 | RETAINED | 228.5 | 48.574 | 220.091 | 55.68 | 180.667 | 39.786 |
| Gly | Glycine | Amino acid | 0.93 | RETAINED | 591.167 | 77.254 | 743.273 | 107.339 | 610.25 | 89.112 |
| His | Histidine | Amino acid | 4.77 | RETAINED | 82.683 | 10.547 | 102.209 | 15.542 | 95.217 | 17.178 |
| Ile | Isoleucine | Amino acid | 1.63 | RETAINED | 134.5 | 16.034 | 139 | 18.858 | 127.642 | 20.63 |
| Leu | Leucine | Amino acid | 0.7 | RETAINED | 227.417 | 24.723 | 239.364 | 29.361 | 216.417 | 24.622 |
| Lys | Lysine | Amino acid | 2.88 | RETAINED | 185.25 | 25.668 | 216.182 | 36.851 | 202.75 | 34.665 |
| Met | Methionine | Amino acid | 1.32 | RETAINED | 36.042 | 7.952 | 43.082 | 6.458 | 35.45 | 5.437 |
| Orn | Ornithine | Amino acid | 2.73 | RETAINED | 61.008 | 7.092 | 74.809 | 15.344 | 64.742 | 9.714 |
| Phe | Phenylalanine | Amino acid | 4.43 | RETAINED | 81.125 | 8.971 | 85.018 | 8.073 | 82.875 | 10.25 |
| Pro | Proline | Amino acid | 4.28 | RETAINED | 161.75 | 19.088 | 212.364 | 29.607 | 195.917 | 34.473 |
| Ser | Serine | Amino acid | 6.03 | RETAINED | 95.475 | 10.196 | 107.964 | 16.176 | 95.883 | 13.166 |
| Thr | Threonine | Amino acid | 5.6 | RETAINED | 118.317 | 23.458 | 152.818 | 26.097 | 122.4 | 17.734 |
| Trp | Tryptophan | Amino acid | 7.01 | RETAINED | 64.908 | 8.859 | 61.618 | 10.475 | 52.425 | 8.64 |
| Tyr | Tyrosine | Amino acid | 2.8 | RETAINED | 70.95 | 12.325 | 82.027 | 11.533 | 73.975 | 12.029 |
| Val | Valine | Amino acid | 3.86 | RETAINED | 246.083 | 23.099 | 254.364 | 27.828 | 237.917 | 24.868 |
| ADMA | Asymmetric dimethylarginine | Biogenic amine | 17.56 | RETAINED | 0.93 | 0.181 | 1.325 | 0.162 | 1.36 | 0.361 |
| Ac-Orn | Acetylornithine | Biogenic amine | 11.7 | RETAINED | 5.803 | 2.125 | 12.015 | 3.109 | 10.538 | 2.854 |
| Carnosine | Carnosine | Biogenic amine | 19.83 | RETAINED | 36.05 | 7.617 | 49.191 | 16.607 | 41.1 | 16.088 |
| Creatinine | Creatinine | Biogenic amine | 5.46 | RETAINED | 152.667 | 16.755 | 167.545 | 25.087 | 173.167 | 23.455 |
| Histamine | Histamine | Biogenic amine | 19.42 | RETAINED | 0.278 | 0.36 | 0.435 | 0.565 | 0.685 | 1.011 |
| Kynurenine | Kynurenine | Biogenic amine | 0.21 | RETAINED | 0.447 | 0.32 | 0.916 | 0.43 | 1.024 | 0.255 |
| Met-SO | Methioninesulfoxide | Biogenic amine | 3.73 | RETAINED | 0.876 | 0.269 | 1.095 | 0.243 | 0.937 | 0.259 |
| Nitro-Tyr | 3-nitrotyrosine | Biogenic amine | NA | DISCARDED | 0 | 0 | 0 | 0 | 0 | 0 |
| OH-Pro | Hydroxyproline | Biogenic amine | NA | DISCARDED | NA | NA | NA | NA | NA | NA |
| PEA | Phenylethylamine | Biogenic amine | NA | DISCARDED | 0 | 0 | 0 | 0 | 0 | 0 |
| Putrescine | Putrescine | Biogenic amine | 15.03 | RETAINED | 0.608 | 0.212 | 0.65 | 0.193 | 0.679 | 0.19 |
| SDMA | Symmetric Dimethylarginine | Biogenic amine | 18.7 | RETAINED | 0.019 | 0.038 | 0.072 | 0.137 | 0.095 | 0.163 |
| Sarcosine | Sarcosine | Biogenic amine | 19.42 | RETAINED | 9.063 | 2.209 | 8.276 | 2.39 | 8.831 | 1.896 |
| Serotonin | Serotonin | Biogenic amine | 7.48 | RETAINED | 0.861 | 0.566 | 1.226 | 0.755 | 1.329 | 1.065 |
| Spermidine | Spermidine | Biogenic amine | 4.23 | RETAINED | 0.782 | 0.372 | 0.818 | 0.329 | 0.938 | 0.307 |
| Spermine | Spermine | Biogenic amine | 1.07 | RETAINED | 0.622 | 0.319 | 0.503 | 0.071 | 0.66 | 0.185 |
| Taurine | Taurine | Biogenic amine | 4.38 | RETAINED | 133.767 | 32.348 | 136.818 | 26.02 | 142.45 | 33.375 |
| alpha-AAA | alpha-Aminoadipic acid | Biogenic amine | 18.1 | RETAINED | 38.45 | 6.213 | 29.09 | 13.992 | 29.725 | 7.876 |
| total DMA | Sum of ADMA and SDMA | Biogenic amine | 7.58 | RETAINED | 0.925 | 0.219 | 1.152 | 0.21 | 1.253 | 0.314 |
| PC aa C24:0 | Phosphatidylcholine diacyl C24:0 | Glycerophospholipid | 17.83 | RETAINED | 0.7 | 0.214 | 1.212 | 0.337 | 0.902 | 0.165 |
| PC aa C26:0 | Phosphatidylcholine diacyl C26:0 | Glycerophospholipid | 16.39 | RETAINED | 1.49 | 0.326 | 2.315 | 0.542 | 1.797 | 0.312 |
| PC aa C28:1 | Phosphatidylcholine diacyl C28:1 | Glycerophospholipid | 8.15 | RETAINED | 0.621 | 0.098 | 0.769 | 0.172 | 0.688 | 0.109 |
| PC aa C30:0 | Phosphatidylcholine diacyl C30:0 | Glycerophospholipid | 9.84 | RETAINED | 1.244 | 0.24 | 1.257 | 0.199 | 1.107 | 0.188 |
| PC aa C30:2 | Phosphatidylcholine diacyl C30:2 | Glycerophospholipid | 2.75 | RETAINED | 0.495 | 0.091 | 0.583 | 0.101 | 0.506 | 0.096 |
| PC aa C32:0 | Phosphatidylcholine diacyl C32:0 | Glycerophospholipid | 6.4 | RETAINED | 4.066 | 0.815 | 4.75 | 1 | 4.02 | 0.628 |
| PC aa C32:1 | Phosphatidylcholine diacyl C32:1 | Glycerophospholipid | 5.27 | RETAINED | 1.733 | 0.301 | 2.647 | 0.586 | 1.846 | 0.317 |
| PC aa C32:2 | Phosphatidylcholine diacyl C32:2 | Glycerophospholipid | 8.31 | RETAINED | 0.563 | 0.056 | 0.654 | 0.099 | 0.585 | 0.081 |
| PC aa C32:3 | Phosphatidylcholine diacyl C32:3 | Glycerophospholipid | 10.43 | RETAINED | 0.133 | 0.024 | 0.171 | 0.03 | 0.146 | 0.019 |
| PC aa C34:1 | Phosphatidylcholine diacyl C34:1 | Glycerophospholipid | 6.43 | RETAINED | 65.15 | 8.848 | 87.009 | 13.671 | 70.342 | 11.797 |
| PC aa C34:2 | Phosphatidylcholine diacyl C34:2 | Glycerophospholipid | 6.94 | RETAINED | 78.733 | 8.836 | 98.027 | 18.872 | 79.083 | 12.582 |
| PC aa C34:3 | Phosphatidylcholine diacyl C34:3 | Glycerophospholipid | 5.44 | RETAINED | 2.96 | 0.388 | 3.735 | 0.713 | 2.963 | 0.564 |
| PC aa C34:4 | Phosphatidylcholine diacyl C34:4 | Glycerophospholipid | 5.71 | RETAINED | 0.191 | 0.024 | 0.252 | 0.039 | 0.21 | 0.038 |
| PC aa C36:0 | Phosphatidylcholine diacyl C36:0 | Glycerophospholipid | 4.83 | RETAINED | 0.651 | 0.148 | 0.946 | 0.237 | 0.706 | 0.158 |
| PC aa C36:1 | Phosphatidylcholine diacyl C36:1 | Glycerophospholipid | 7.25 | RETAINED | 37.442 | 4.978 | 47.273 | 8.498 | 37.15 | 7.911 |
| PC aa C36:2 | Phosphatidylcholine diacyl C36:2 | Glycerophospholipid | 6.52 | RETAINED | 97.367 | 10.145 | 114.718 | 18.503 | 92.983 | 15.347 |
| PC aa C36:3 | Phosphatidylcholine diacyl C36:3 | Glycerophospholipid | 7.86 | RETAINED | 18.833 | 1.524 | 24.518 | 5.412 | 18.642 | 3.05 |
| PC aa C36:4 | Phosphatidylcholine diacyl C36:4 | Glycerophospholipid | 6.21 | RETAINED | 42.058 | 6.301 | 61.173 | 9.849 | 45.433 | 11.318 |
| PC aa C36:5 | Phosphatidylcholine diacyl C36:5 | Glycerophospholipid | 5.5 | RETAINED | 1.659 | 0.294 | 2.746 | 0.559 | 1.643 | 0.421 |
| PC aa C36:6 | Phosphatidylcholine diacyl C36:6 | Glycerophospholipid | 4 | RETAINED | 0.11 | 0.022 | 0.142 | 0.024 | 0.111 | 0.021 |
| PC aa C38:0 | Phosphatidylcholine diacyl C38:0 | Glycerophospholipid | 7.69 | RETAINED | 0.417 | 0.093 | 0.469 | 0.087 | 0.399 | 0.079 |
| PC aa C38:1 | Phosphatidylcholine diacyl C38:1 | Glycerophospholipid | 0.29 | RETAINED | 0.456 | 0.139 | 0.533 | 0.114 | 0.453 | 0.121 |
| PC aa C38:3 | Phosphatidylcholine diacyl C38:3 | Glycerophospholipid | 5.7 | RETAINED | 18.908 | 2.66 | 25.391 | 4.624 | 18.533 | 3.694 |
| PC aa C38:4 | Phosphatidylcholine diacyl C38:4 | Glycerophospholipid | 5.79 | RETAINED | 126.55 | 18.608 | 153 | 15.118 | 134.717 | 28.78 |
| PC aa C38:5 | Phosphatidylcholine diacyl C38:5 | Glycerophospholipid | 7.25 | RETAINED | 25.333 | 3.46 | 33.436 | 4.186 | 27.083 | 6.345 |
| PC aa C38:6 | Phosphatidylcholine diacyl C38:6 | Glycerophospholipid | 6.06 | RETAINED | 10.818 | 2.555 | 14.484 | 3.652 | 10.809 | 3.381 |
| PC aa C40:1 | Phosphatidylcholine diacyl C40:1 | Glycerophospholipid | 4.76 | RETAINED | 0.24 | 0.029 | 0.283 | 0.037 | 0.257 | 0.019 |
| PC aa C40:2 | Phosphatidylcholine diacyl C40:2 | Glycerophospholipid | 6.52 | RETAINED | 0.239 | 0.047 | 0.351 | 0.066 | 0.28 | 0.058 |
| PC aa C40:3 | Phosphatidylcholine diacyl C40:3 | Glycerophospholipid | 5.07 | RETAINED | 0.39 | 0.07 | 0.505 | 0.079 | 0.427 | 0.066 |
| PC aa C40:4 | Phosphatidylcholine diacyl C40:4 | Glycerophospholipid | 7.83 | RETAINED | 7.483 | 1.465 | 10.435 | 3.249 | 9.698 | 2.316 |
| PC aa C40:5 | Phosphatidylcholine diacyl C40:5 | Glycerophospholipid | 5.9 | RETAINED | 19.908 | 3.118 | 24.018 | 4.62 | 21.975 | 5.269 |
| PC aa C40:6 | Phosphatidylcholine diacyl C40:6 | Glycerophospholipid | 6.56 | RETAINED | 14.05 | 3.411 | 17.6 | 5.418 | 13.274 | 4.152 |
| PC aa C42:0 | Phosphatidylcholine diacyl C42:0 | Glycerophospholipid | 4.74 | RETAINED | 0.12 | 0.024 | 0.154 | 0.031 | 0.134 | 0.024 |
| PC aa C42:1 | Phosphatidylcholine diacyl C42:1 | Glycerophospholipid | 7 | RETAINED | 0.084 | 0.018 | 0.11 | 0.023 | 0.09 | 0.015 |
| PC aa C42:2 | Phosphatidylcholine diacyl C42:2 | Glycerophospholipid | 6.76 | RETAINED | 0.125 | 0.021 | 0.158 | 0.027 | 0.127 | 0.017 |
| PC aa C42:4 | Phosphatidylcholine diacyl C42:4 | Glycerophospholipid | 4.02 | RETAINED | 0.143 | 0.031 | 0.197 | 0.037 | 0.151 | 0.027 |
| PC aa C42:5 | Phosphatidylcholine diacyl C42:5 | Glycerophospholipid | 9.15 | RETAINED | 0.235 | 0.072 | 0.319 | 0.082 | 0.234 | 0.058 |
| PC aa C42:6 | Phosphatidylcholine diacyl C42:6 | Glycerophospholipid | 10.35 | RETAINED | 0.577 | 0.088 | 0.698 | 0.122 | 0.592 | 0.057 |
| PC ae C30:0 | Phosphatidylcholine acyl-alkyl C30:0 | Glycerophospholipid | 9.99 | RETAINED | 0.259 | 0.039 | 0.296 | 0.044 | 0.262 | 0.03 |
| PC ae C30:1 | Phosphatidylcholine acyl-alkyl C30:1 | Glycerophospholipid | 18.18 | RETAINED | 0.251 | 0.063 | 0.396 | 0.099 | 0.3 | 0.068 |
| PC ae C30:2 | Phosphatidylcholine acyl-alkyl C30:2 | Glycerophospholipid | 9.84 | RETAINED | 0.106 | 0.03 | 0.147 | 0.03 | 0.121 | 0.021 |
| PC ae C32:1 | Phosphatidylcholine acyl-alkyl C32:1 | Glycerophospholipid | 11.22 | RETAINED | 1.026 | 0.187 | 1.2 | 0.253 | 1.044 | 0.146 |
| PC ae C32:2 | Phosphatidylcholine acyl-alkyl C32:2 | Glycerophospholipid | 9.62 | RETAINED | 0.292 | 0.042 | 0.324 | 0.068 | 0.285 | 0.036 |
| PC ae C34:0 | Phosphatidylcholine acyl-alkyl C34:0 | Glycerophospholipid | 7.87 | RETAINED | 0.481 | 0.103 | 0.506 | 0.107 | 0.457 | 0.068 |
| PC ae C34:1 | Phosphatidylcholine acyl-alkyl C34:1 | Glycerophospholipid | 4.45 | RETAINED | 3.868 | 0.584 | 4.508 | 0.812 | 4.094 | 0.663 |
| PC ae C34:2 | Phosphatidylcholine acyl-alkyl C34:2 | Glycerophospholipid | 2.74 | RETAINED | 3.899 | 0.682 | 4.455 | 0.795 | 4.454 | 0.798 |
| PC ae C34:3 | Phosphatidylcholine acyl-alkyl C34:3 | Glycerophospholipid | 3.85 | RETAINED | 2.184 | 0.465 | 2.116 | 0.343 | 2.085 | 0.455 |
| PC ae C36:0 | Phosphatidylcholine acyl-alkyl C36:0 | Glycerophospholipid | 2.87 | RETAINED | 0.225 | 0.041 | 0.27 | 0.029 | 0.219 | 0.036 |
| PC ae C36:1 | Phosphatidylcholine acyl-alkyl C36:1 | Glycerophospholipid | 3.83 | RETAINED | 3.154 | 0.414 | 3.943 | 0.778 | 3.09 | 0.631 |
| PC ae C36:2 | Phosphatidylcholine acyl-alkyl C36:2 | Glycerophospholipid | 5.45 | RETAINED | 5.146 | 0.632 | 5.957 | 1.135 | 4.897 | 0.874 |
| PC ae C36:3 | Phosphatidylcholine acyl-alkyl C36:3 | Glycerophospholipid | 5.68 | RETAINED | 2.78 | 0.489 | 3.057 | 0.589 | 2.971 | 0.637 |
| PC ae C36:4 | Phosphatidylcholine acyl-alkyl C36:4 | Glycerophospholipid | 7.12 | RETAINED | 2.802 | 0.543 | 3.038 | 0.647 | 3.063 | 0.664 |
| PC ae C36:5 | Phosphatidylcholine acyl-alkyl C36:5 | Glycerophospholipid | 6.21 | RETAINED | 1.242 | 0.254 | 1.357 | 0.279 | 1.293 | 0.302 |
| PC ae C38:0 | Phosphatidylcholine acyl-alkyl C38:0 | Glycerophospholipid | 8.11 | RETAINED | 0.389 | 0.069 | 0.516 | 0.089 | 0.402 | 0.08 |
| PC ae C38:1 | Phosphatidylcholine acyl-alkyl C38:1 | Glycerophospholipid | 6.98 | RETAINED | 0.53 | 0.071 | 0.647 | 0.13 | 0.507 | 0.106 |
| PC ae C38:2 | Phosphatidylcholine acyl-alkyl C38:2 | Glycerophospholipid | 6.22 | RETAINED | 0.971 | 0.131 | 1.081 | 0.162 | 0.907 | 0.145 |
| PC ae C38:3 | Phosphatidylcholine acyl-alkyl C38:3 | Glycerophospholipid | 4.96 | RETAINED | 1.136 | 0.163 | 1.355 | 0.224 | 1.046 | 0.158 |
| PC ae C38:4 | Phosphatidylcholine acyl-alkyl C38:4 | Glycerophospholipid | 5.49 | RETAINED | 6.326 | 1.102 | 7.58 | 1.21 | 6.538 | 1.483 |
| PC ae C38:5 | Phosphatidylcholine acyl-alkyl C38:5 | Glycerophospholipid | 6.88 | RETAINED | 3.42 | 0.581 | 3.831 | 0.742 | 3.708 | 0.82 |
| PC ae C38:6 | Phosphatidylcholine acyl-alkyl C38:6 | Glycerophospholipid | 6.75 | RETAINED | 0.75 | 0.146 | 0.791 | 0.165 | 0.747 | 0.15 |
| PC ae C40:1 | Phosphatidylcholine acyl-alkyl C40:1 | Glycerophospholipid | 5.29 | RETAINED | 0.663 | 0.16 | 1.171 | 0.23 | 0.702 | 0.166 |
| PC ae C40:2 | Phosphatidylcholine acyl-alkyl C40:2 | Glycerophospholipid | 6.28 | RETAINED | 0.349 | 0.043 | 0.417 | 0.057 | 0.347 | 0.039 |
| PC ae C40:3 | Phosphatidylcholine acyl-alkyl C40:3 | Glycerophospholipid | 5.26 | RETAINED | 0.422 | 0.079 | 0.519 | 0.088 | 0.442 | 0.073 |
| PC ae C40:4 | Phosphatidylcholine acyl-alkyl C40:4 | Glycerophospholipid | 6.28 | RETAINED | 1.223 | 0.19 | 1.456 | 0.226 | 1.293 | 0.228 |
| PC ae C40:5 | Phosphatidylcholine acyl-alkyl C40:5 | Glycerophospholipid | 5.67 | RETAINED | 1.647 | 0.265 | 1.88 | 0.315 | 1.687 | 0.319 |
| PC ae C40:6 | Phosphatidylcholine acyl-alkyl C40:6 | Glycerophospholipid | 6.59 | RETAINED | 1.012 | 0.208 | 1.135 | 0.259 | 0.973 | 0.247 |
| PC ae C42:0 | Phosphatidylcholine acyl-akyl C42:0 | Glycerophospholipid | 4.73 | RETAINED | 0.463 | 0.037 | 0.537 | 0.059 | 0.48 | 0.033 |
| PC ae C42:1 | Phosphatidylcholine acyl-alkyl C42:1 | Glycerophospholipid | 5.78 | RETAINED | 0.747 | 0.215 | 1.282 | 0.285 | 0.939 | 0.193 |
| PC ae C42:2 | Phosphatidylcholine acyl-alkyl C42:2 | Glycerophospholipid | 9.46 | RETAINED | 0.318 | 0.061 | 0.546 | 0.103 | 0.351 | 0.071 |
| PC ae C42:3 | Phosphatidylcholine acyl-alkyl C42:3 | Glycerophospholipid | 9.09 | RETAINED | 0.202 | 0.047 | 0.3 | 0.067 | 0.213 | 0.04 |
| PC ae C42:4 | Phosphatidylcholine acyl-alkyl C42:4 | Glycerophospholipid | 5.69 | RETAINED | 0.192 | 0.038 | 0.227 | 0.055 | 0.208 | 0.033 |
| PC ae C42:5 | Phosphatidylcholine acyl-alkyl C42:5 | Glycerophospholipid | 7.1 | RETAINED | 0.556 | 0.058 | 0.604 | 0.072 | 0.551 | 0.052 |
| PC ae C44:3 | Phosphatidylcholine acyl-alkyl C44:3 | Glycerophospholipid | 6.7 | RETAINED | 0.128 | 0.032 | 0.203 | 0.049 | 0.148 | 0.016 |
| PC ae C44:4 | Phosphatidylcholine acyl-alkyl C44:4 | Glycerophospholipid | 8.65 | RETAINED | 0.119 | 0.021 | 0.156 | 0.029 | 0.121 | 0.013 |
| PC ae C44:5 | Phosphatidylcholine acyl-alkyl C44:5 | Glycerophospholipid | 6.98 | RETAINED | 0.198 | 0.041 | 0.236 | 0.056 | 0.199 | 0.035 |
| PC ae C44:6 | Phosphatidylcholine acyl-alkyl C44:6 | Glycerophospholipid | 10.8 | RETAINED | 0.193 | 0.041 | 0.243 | 0.067 | 0.204 | 0.042 |
| lysoPC a C14:0 | lysoPhosphatidylcholine acyl C14:0 | Glycerophospholipid | 5.15 | RETAINED | 2.325 | 0.179 | 2.525 | 0.116 | 2.578 | 0.154 |
| lysoPC a C16:0 | lysoPhosphatidylcholine acyl C16:0 | Glycerophospholipid | 7.31 | RETAINED | 45.867 | 5.013 | 57.564 | 9.196 | 51.042 | 7.28 |
| lysoPC a C16:1 | lysoPhosphatidylcholine acyl C16:1 | Glycerophospholipid | 6.9 | RETAINED | 1.258 | 0.176 | 1.495 | 0.145 | 1.394 | 0.199 |
| lysoPC a C17:0 | lysoPhosphatidylcholine acyl C17:0 | Glycerophospholipid | 7.62 | RETAINED | 1.297 | 0.204 | 1.615 | 0.353 | 1.318 | 0.286 |
| lysoPC a C18:0 | lysoPhosphatidylcholine acyl C18:0 | Glycerophospholipid | 5.63 | RETAINED | 34.633 | 3.184 | 42.218 | 7.231 | 37.008 | 5.974 |
| lysoPC a C18:1 | lysoPhosphatidylcholine acyl C18:1 | Glycerophospholipid | 4.72 | RETAINED | 16.025 | 3.199 | 19.127 | 2.819 | 15.875 | 2.784 |
| lysoPC a C18:2 | lysoPhosphatidylcholine acyl C18:2 | Glycerophospholipid | 4.44 | RETAINED | 18.042 | 3.795 | 20.6 | 3.496 | 15.9 | 2.934 |
| lysoPC a C20:3 | lysoPhosphatidylcholine acyl C20:3 | Glycerophospholipid | 6.82 | RETAINED | 1.01 | 0.206 | 1.186 | 0.234 | 0.787 | 0.147 |
| lysoPC a C20:4 | lysoPhosphatidylcholine acyl C20:4 | Glycerophospholipid | 2.7 | RETAINED | 9.431 | 2.796 | 11 | 2.287 | 8.478 | 2.562 |
| lysoPC a C24:0 | lysoPhosphatidylcholine acyl C24:0 | Glycerophospholipid | 19.4 | RETAINED | 0.505 | 0.172 | 0.8 | 0.195 | 0.588 | 0.121 |
| lysoPC a C26:0 | lysoPhosphatidylcholine acyl C26:0 | Glycerophospholipid | 18.1 | RETAINED | 0.655 | 0.199 | 1.051 | 0.254 | 0.804 | 0.163 |
| lysoPC a C26:1 | lysoPhosphatidylcholine acyl C26:1 | Glycerophospholipid | 14.06 | RETAINED | 0.362 | 0.088 | 0.588 | 0.144 | 0.448 | 0.091 |
| lysoPC a C28:0 | lysoPhosphatidylcholine acyl C28:0 | Glycerophospholipid | 19.73 | RETAINED | 0.914 | 0.281 | 1.361 | 0.379 | 0.984 | 0.152 |
| lysoPC a C28:1 | lysoPhosphatidylcholine acyl C28:1 | Glycerophospholipid | 12.31 | RETAINED | 1.165 | 0.387 | 1.854 | 0.517 | 1.452 | 0.283 |
| SM (OH) C14:1 | Hydroxysphingomyeline C14:1 | Sphingomyelin | 6.71 | RETAINED | 1.544 | 0.184 | 1.491 | 0.226 | 1.28 | 0.2 |
| SM (OH) C16:1 | Hydroxysphingomyeline C16:1 | Sphingomyelin | 11.11 | RETAINED | 2.728 | 0.302 | 2.462 | 0.389 | 2.261 | 0.446 |
| SM (OH) C22:1 | Hydroxysphingomyeline C22:1 | Sphingomyelin | 10.17 | RETAINED | 3.873 | 0.449 | 3.508 | 0.413 | 3.594 | 0.669 |
| SM (OH) C22:2 | Hydroxysphingomyeline C22:2 | Sphingomyelin | 7.5 | RETAINED | 1.514 | 0.187 | 1.313 | 0.19 | 1.245 | 0.215 |
| SM (OH) C24:1 | Hydroxysphingomyeline C24:1 | Sphingomyelin | 6.52 | RETAINED | 0.481 | 0.067 | 0.585 | 0.088 | 0.501 | 0.065 |
| SM C16:0 | Sphingomyeline C16:0 | Sphingomyelin | 7.75 | RETAINED | 66.292 | 7.747 | 60.518 | 7.902 | 59.35 | 9.123 |
| SM C16:1 | Sphingomyeline C16:1 | Sphingomyelin | 7.46 | RETAINED | 3.614 | 0.564 | 3.53 | 0.802 | 3.3 | 0.621 |
| SM C18:0 | Sphingomyeline C18:0 | Sphingomyelin | 9.14 | RETAINED | 17.492 | 2.64 | 15.382 | 2.259 | 17.092 | 3.164 |
| SM C18:1 | Sphingomyeline C18:1 | Sphingomyelin | 6.43 | RETAINED | 3.6 | 0.547 | 3.043 | 0.633 | 3.273 | 0.628 |
| SM C20:2 | Sphingomyeline C20:2 | Sphingomyelin | 9.67 | RETAINED | 0.427 | 0.053 | 0.454 | 0.075 | 0.415 | 0.065 |
| SM C22:3 | Sphingomyeline C22:3 | Sphingomyelin | 8.81 | RETAINED | 3.08 | 0.535 | 4.768 | 1.06 | 3.391 | 0.948 |
| SM C24:0 | Sphingomyeline C24:0 | Sphingomyelin | 9.14 | RETAINED | 12.206 | 1.541 | 13.299 | 1.699 | 11.191 | 1.409 |
| SM C24:1 | Sphingomyeline C24:1 | Sphingomyelin | 9.41 | RETAINED | 18.325 | 2.504 | 15.273 | 1.914 | 15.733 | 2.327 |
| SM C26:0 | Sphingomyeline C26:0 | Sphingomyelin | 19.97 | RETAINED | 0.092 | 0.029 | 0.107 | 0.025 | 0.107 | 0.032 |
| SM C26:1 | Sphingomyeline C26:1 | Sphingomyelin | 9.9 | RETAINED | 0.179 | 0.047 | 0.184 | 0.028 | 0.182 | 0.035 |
| H1 | Hexose | Sugars | 7.34 | RETAINED | 6397 | 1081 | 5530 | 782 | 5197 | 940 |

**Table S2**. Percentage (%) of Boruta runs in which each of the 22 identified metabolites was selected in pairwise and multiclass analyses across the three pig breeds (Italian Large White, ILW; Italian Landrace, ILA; Italian Duroc, IDU).

| **Metabolite** | **Biochemical name** | **Metabolite class** | **ILA-IDU**  **Runs (%)** | **ILW-ILA**  **Runs (%)** | **ILW-IDU**  **Runs (%)** | **Multi-class**  **Runs (%)** |
| --- | --- | --- | --- | --- | --- | --- |
| Trp | Tryptophan | Amino acid |  |  | 96 |  |
| Thr | Threonine | Amino acid | 100 | 100 |  |  |
| SM C22:3 | Sphingomyeline C22:3 | Sphingomyelin | 100 |  |  |  |
| SM (OH) C22:2 | Hydroxysphingomyeline C22:2 | Sphingomyelin |  |  | 100 | 100 |
| SM (OH) C16:1 | Hydroxysphingomyeline C16:1 | Sphingomyelin |  |  | 98 |  |
| SM (OH) C14:1 | Hydroxysphingomyeline C14:1 | Sphingomyelin |  |  | 100 | 95 |
| Pro | Proline | Amino acid | 100 |  |  |  |
| PC ae C44:3 | Phosphatidylcholine acyl-alkyl C44:3 | Glycerophospholipid |  |  | 91 |  |
| PC ae C42:2 | Phosphatidylcholine acyl-alkyl C42:2 | Glycerophospholipid | 100 | 100 |  | 100 |
| PC ae C40:1 | Phosphatidylcholine acyl-alkyl C40:1 | Glycerophospholipid | 100 | 99 |  | 98 |
| PC aa C38:5 | Phosphatidylcholine diacyl C38:5 | Glycerophospholipid | 100 |  |  |  |
| PC aa C36:5 | Phosphatidylcholine diacyl C36:5 | Glycerophospholipid | 100 | 97 |  | 100 |
| PC aa C36:4 | Phosphatidylcholine diacyl C36:4 | Glycerophospholipid | 100 |  |  |  |
| lysoPC a C20:3 | lysoPhosphatidylcholine acyl C20:3 | Glycerophospholipid |  | 100 | 96 | 100 |
| lysoPC a C14:0 | lysoPhosphatidylcholine acyl C14:0 | Glycerophospholipid |  |  | 91 |  |
| Kynurenine | Kynurenine | Biogenic amine | 100 |  | 100 | 100 |
| C6:1 | Hexenoylcarnitine | Acylcarnitine | 98 |  |  | 87 |
| C6 (C4:1-DC) | Hexanoylcarnitine | Acylcarnitine | 100 |  |  | 91 |
| C3-DC (C4-OH) | Hydroxybutyrylcarnitine | Acylcarnitine | 94 |  |  |  |
| C18:2 | Octadecadienylcarnitine | Acylcarnitine |  |  | 97 |  |
| ADMA | Asymmetric dimethylarginine | Biogenic amine | 95 |  | 100 | 81 |
| Ac-Orn | Acetylornithine | Biogenic amine | 100 |  | 100 | 100 |

**Table S3**. Pearson correlation coefficients (lower triangle) and corresponding *p*-values (upper triangle) for the 22 metabolites identified by Boruta in pairwise comparisons of Italian Landrace pigs.

|  | **C18:2** | **C3-DC (C4-OH)** | **C6 (C4:1-DC)** | **C6:1** | **Pro** | **Thr** | **Trp** | **ADMA** | **Ac-Orn** | **Kynurenine** | **PC aa C36:4** | **PC aa C36:5** | **PC aa C38:5** | **PC ae C40:1** | **PC ae C42:2** | **PC ae C44:3** | **lysoPC a C14:0** | **lysoPC a C20:3** | **SM (OH) C14:1** | **SM (OH) C16:1** | **SM (OH) C22:2** | **SM C22:3** |
| --- | --- | --- | --- | --- | --- | --- | --- | --- | --- | --- | --- | --- | --- | --- | --- | --- | --- | --- | --- | --- | --- | --- |
| **C18:2** |  | 8.1E-02 | 3.2E-02 | 2.6E-01 | 4.0E-01 | 8.0E-01 | 1.1E-01 | 7.8E-01 | 1.0E+00 | 6.5E-01 | 9.1E-01 | 8.4E-01 | 9.0E-01 | 9.1E-01 | 9.0E-01 | 4.1E-01 | 9.3E-01 | 3.0E-01 | 6.8E-01 | 2.8E-01 | 8.0E-01 | 9.1E-01 |
| **C3-DC (C4-OH)** | 5.5E-01 |  | 2.8E-02 | 7.6E-01 | 8.9E-01 | 5.5E-01 | 2.4E-02 | 7.3E-01 | 8.9E-01 | 3.0E-01 | 4.0E-01 | 4.7E-01 | 2.9E-01 | 4.9E-01 | 4.0E-01 | 8.0E-01 | 8.8E-01 | 3.0E-01 | 1.7E-01 | 8.6E-01 | 6.9E-01 | 3.6E-01 |
| **C6 (C4:1-DC)** | 6.4E-01 | 6.6E-01 |  | 7.7E-01 | 1.5E-01 | 7.7E-01 | 4.0E-01 | 8.0E-01 | 5.8E-01 | 4.8E-01 | 5.0E-01 | 6.7E-01 | 6.7E-01 | 3.0E-01 | 6.8E-01 | 5.8E-02 | 9.6E-01 | 8.2E-01 | 1.0E+00 | 3.1E-01 | 9.5E-01 | 4.7E-01 |
| **C6:1** | -3.8E-01 | 1.0E-01 | -1.0E-01 |  | 1.6E-02 | 6.4E-01 | 6.5E-01 | 4.0E-01 | 3.8E-01 | 7.4E-01 | 2.2E-01 | 8.5E-02 | 2.8E-01 | 9.3E-01 | 8.5E-01 | 8.1E-01 | 5.8E-01 | 9.3E-01 | 9.5E-01 | 8.7E-01 | 7.0E-01 | 3.4E-01 |
| **Pro** | -2.8E-01 | 4.6E-02 | -4.7E-01 | 7.0E-01 |  | 7.6E-01 | 9.1E-01 | 6.0E-01 | 1.2E-01 | 1.0E+00 | 1.1E-02 | 3.1E-02 | 3.9E-02 | 3.8E-01 | 4.5E-01 | 3.2E-01 | 1.7E-01 | 9.6E-01 | 4.0E-01 | 3.9E-01 | 6.8E-01 | 2.0E-02 |
| **Thr** | 8.6E-02 | 2.0E-01 | -9.9E-02 | 1.6E-01 | 1.0E-01 |  | 7.8E-01 | 3.4E-02 | 1.9E-01 | 9.5E-01 | 6.8E-01 | 5.2E-01 | 3.0E-01 | 1.1E-01 | 4.0E-02 | 3.4E-01 | 7.9E-01 | 2.5E-01 | 4.7E-01 | 5.9E-01 | 4.7E-01 | 7.1E-01 |
| **Trp** | 5.1E-01 | 6.7E-01 | 2.8E-01 | -1.5E-01 | 4.1E-02 | 9.4E-02 |  | 5.8E-01 | 8.2E-01 | 9.9E-02 | 8.3E-01 | 6.7E-01 | 8.1E-01 | 5.4E-01 | 7.2E-01 | 7.8E-01 | 4.8E-01 | 3.5E-01 | 5.4E-01 | 5.7E-01 | 6.7E-01 | 7.4E-01 |
| **ADMA** | -9.7E-02 | 1.2E-01 | 8.8E-02 | 2.8E-01 | 1.8E-01 | 6.4E-01 | -1.9E-01 |  | 2.1E-01 | 8.2E-01 | 4.6E-01 | 5.4E-01 | 2.3E-01 | 3.0E-01 | 4.6E-02 | 3.6E-01 | 1.9E-01 | 9.4E-01 | 2.4E-01 | 8.0E-01 | 9.6E-01 | 5.3E-01 |
| **Ac-Orn** | -1.4E-03 | 4.7E-02 | -1.9E-01 | 2.9E-01 | 5.0E-01 | -4.3E-01 | -7.6E-02 | -4.1E-01 |  | 7.3E-01 | 2.4E-01 | 4.5E-01 | 6.7E-01 | 8.5E-01 | 3.7E-01 | 7.8E-01 | 8.4E-01 | 7.2E-01 | 8.2E-01 | 7.0E-01 | 6.3E-01 | 2.8E-01 |
| **Kynurenine** | 1.6E-01 | 3.5E-01 | 2.4E-01 | -1.2E-01 | 1.3E-04 | -2.1E-02 | 5.2E-01 | -7.7E-02 | 1.2E-01 |  | 8.2E-01 | 2.3E-01 | 5.5E-01 | 4.1E-01 | 4.4E-01 | 4.9E-01 | 9.0E-01 | 9.2E-01 | 7.4E-01 | 8.0E-01 | 7.5E-02 | 7.6E-01 |
| **PC aa C36:4** | -3.7E-02 | 2.8E-01 | -2.3E-01 | 4.0E-01 | 7.3E-01 | 1.4E-01 | 7.3E-02 | 2.5E-01 | 3.9E-01 | -7.7E-02 |  | 1.0E-03 | 7.0E-06 | 7.6E-02 | 2.5E-02 | 3.0E-02 | 1.3E-02 | 1.0E-01 | 2.6E-03 | 1.0E-01 | 5.5E-02 | 8.1E-09 |
| **PC aa C36:5** | -6.9E-02 | 2.4E-01 | -1.5E-01 | 5.4E-01 | 6.5E-01 | 2.2E-01 | -1.4E-01 | 2.1E-01 | 2.6E-01 | -4.0E-01 | 8.5E-01 |  | 2.3E-04 | 2.4E-01 | 5.9E-02 | 1.9E-01 | 3.3E-02 | 1.1E-01 | 5.5E-02 | 5.5E-01 | 5.2E-02 | 1.4E-03 |
| **PC aa C38:5** | 4.1E-02 | 3.5E-01 | -1.5E-01 | 3.6E-01 | 6.3E-01 | 3.4E-01 | 8.0E-02 | 4.0E-01 | 1.5E-01 | -2.0E-01 | 9.5E-01 | 8.9E-01 |  | 4.1E-02 | 1.9E-03 | 2.8E-02 | 1.3E-02 | 5.0E-02 | 1.0E-03 | 1.9E-01 | 4.1E-02 | 6.1E-06 |
| **PC ae C40:1** | 4.0E-02 | 2.3E-01 | -3.4E-01 | 3.2E-02 | 2.9E-01 | 5.1E-01 | 2.1E-01 | 3.5E-01 | 6.5E-02 | -2.8E-01 | 5.6E-01 | 3.9E-01 | 6.2E-01 |  | 3.0E-03 | 1.4E-03 | 9.0E-01 | 2.0E-01 | 5.3E-03 | 5.2E-02 | 1.8E-01 | 4.5E-02 |
| **PC ae C42:2** | 4.3E-02 | 2.8E-01 | -1.4E-01 | 6.6E-02 | 2.5E-01 | 6.3E-01 | 1.2E-01 | 6.1E-01 | -3.0E-01 | -2.6E-01 | 6.7E-01 | 5.8E-01 | 8.2E-01 | 8.0E-01 |  | 5.8E-03 | 1.5E-01 | 4.3E-02 | 7.0E-04 | 1.4E-01 | 1.1E-01 | 1.6E-02 |
| **PC ae C44:3** | -2.8E-01 | -8.5E-02 | -5.9E-01 | -8.1E-02 | 3.3E-01 | 3.2E-01 | -9.3E-02 | 3.0E-01 | 9.5E-02 | -2.3E-01 | 6.5E-01 | 4.3E-01 | 6.6E-01 | 8.3E-01 | 7.7E-01 |  | 2.9E-01 | 2.8E-01 | 5.7E-03 | 2.0E-02 | 8.4E-02 | 1.5E-02 |
| **lysoPC a C14:0** | -3.0E-02 | 5.3E-02 | -1.6E-02 | 1.9E-01 | 4.5E-01 | 9.2E-02 | -2.4E-01 | 4.2E-01 | 6.8E-02 | 4.2E-02 | 7.2E-01 | 6.4E-01 | 7.2E-01 | 4.3E-02 | 4.7E-01 | 3.5E-01 |  | 3.5E-01 | 1.1E-01 | 7.8E-01 | 1.8E-01 | 2.3E-02 |
| **lysoPC a C20:3** | 3.4E-01 | 3.5E-01 | 8.0E-02 | -3.1E-02 | 1.8E-02 | 3.8E-01 | 3.1E-01 | -2.6E-02 | -1.2E-01 | -3.3E-02 | 5.2E-01 | 5.1E-01 | 6.0E-01 | 4.2E-01 | 6.2E-01 | 3.6E-01 | 3.1E-01 |  | 6.2E-02 | 6.7E-01 | 3.9E-01 | 9.9E-02 |
| **SM (OH) C14:1** | 1.4E-01 | 4.5E-01 | 1.4E-04 | 2.1E-02 | 2.8E-01 | 2.5E-01 | 2.1E-01 | 3.8E-01 | 7.9E-02 | -1.1E-01 | 8.1E-01 | 5.9E-01 | 8.5E-01 | 7.7E-01 | 8.6E-01 | 7.7E-01 | 5.1E-01 | 5.8E-01 |  | 2.4E-02 | 1.6E-02 | 9.0E-04 |
| **SM (OH) C16:1** | -3.6E-01 | 6.2E-02 | -3.4E-01 | 5.6E-02 | 2.9E-01 | -1.8E-01 | 1.9E-01 | 8.6E-02 | 1.3E-01 | -8.5E-02 | 5.2E-01 | 2.0E-01 | 4.3E-01 | 6.0E-01 | 4.8E-01 | 6.8E-01 | 9.5E-02 | 1.5E-01 | 6.7E-01 |  | 1.1E-01 | 6.1E-02 |
| **SM (OH) C22:2** | 8.6E-02 | 1.4E-01 | -2.3E-02 | -1.3E-01 | 1.4E-01 | -2.4E-01 | -1.4E-01 | 1.7E-02 | 1.6E-01 | -5.6E-01 | 5.9E-01 | 6.0E-01 | 6.2E-01 | 4.3E-01 | 5.1E-01 | 5.4E-01 | 4.3E-01 | 2.9E-01 | 7.0E-01 | 5.1E-01 |  | 2.8E-02 |
| **SM C22:3** | -3.8E-02 | 3.1E-01 | -2.5E-01 | 3.2E-01 | 6.9E-01 | 1.3E-01 | 1.2E-01 | 2.1E-01 | 3.6E-01 | -1.1E-01 | 9.9E-01 | 8.3E-01 | 9.5E-01 | 6.1E-01 | 7.0E-01 | 7.1E-01 | 6.7E-01 | 5.2E-01 | 8.5E-01 | 5.8E-01 | 6.6E-01 |  |

**Table S4**. Pearson correlation coefficients (lower triangle) and corresponding *p*-values (upper triangle) for the 22 metabolites identified by Boruta in pairwise comparisons of Italian Duroc pigs.

|  | **C18:2** | **C3-DC (C4-OH)** | **C6 (C4:1-DC)** | **C6:1** | **Pro** | **Thr** | **Trp** | **ADMA** | **Ac-Orn** | **Kynurenine** | **PC aa C36:4** | **PC aa C36:5** | **PC aa C38:5** | **PC ae C40:1** | **PC ae C42:2** | **PC ae C44:3** | **lysoPC a C14:0** | **lysoPC a C20:3** | **SM (OH) C14:1** | **SM (OH) C16:1** | **SM (OH) C22:2** | **SM C22:3** |
| --- | --- | --- | --- | --- | --- | --- | --- | --- | --- | --- | --- | --- | --- | --- | --- | --- | --- | --- | --- | --- | --- | --- |
| **C18:2** |  | 3.5E-01 | 5.5E-01 | 4.2E-01 | 5.7E-01 | 3.6E-01 | 4.3E-02 | 6.3E-01 | 4.5E-01 | 6.9E-01 | 9.5E-01 | 8.7E-01 | 7.4E-01 | 6.5E-01 | 9.2E-01 | 4.1E-01 | 8.7E-01 | 7.7E-01 | 8.3E-01 | 1.9E-01 | 2.0E-01 | 9.2E-01 |
| **C3-DC (C4-OH)** | 3.0E-01 |  | 4.7E-01 | 1.6E-01 | 2.0E-01 | 6.6E-02 | 2.6E-02 | 1.8E-01 | 4.1E-01 | 8.4E-01 | 7.9E-01 | 8.7E-01 | 8.6E-01 | 9.2E-01 | 8.7E-01 | 4.1E-01 | 2.0E-01 | 3.4E-01 | 2.1E-01 | 8.9E-01 | 6.7E-01 | 7.0E-01 |
| **C6 (C4:1-DC)** | -1.9E-01 | 2.3E-01 |  | 2.9E-02 | 3.7E-01 | 6.1E-01 | 1.1E-01 | 3.9E-01 | 1.5E-01 | 8.8E-01 | 9.4E-02 | 2.0E-01 | 9.4E-02 | 8.4E-02 | 6.8E-02 | 4.6E-02 | 1.8E-01 | 4.0E-01 | 8.0E-01 | 2.6E-01 | 7.9E-01 | 2.2E-01 |
| **C6:1** | 2.5E-01 | 4.3E-01 | 6.3E-01 |  | 1.6E-01 | 6.7E-01 | 8.6E-02 | 4.3E-01 | 8.2E-02 | 7.9E-01 | 5.4E-02 | 1.5E-01 | 1.6E-01 | 6.6E-02 | 1.9E-02 | 6.5E-02 | 5.5E-02 | 3.1E-01 | 9.1E-01 | 7.3E-02 | 2.4E-01 | 9.5E-02 |
| **Pro** | 1.8E-01 | 4.0E-01 | 2.9E-01 | 4.3E-01 |  | 1.6E-02 | 1.8E-01 | 1.8E-01 | 1.6E-01 | 6.4E-01 | 1.5E-01 | 1.0E-01 | 3.8E-01 | 8.0E-02 | 1.3E-01 | 7.6E-01 | 1.2E-02 | 6.8E-01 | 2.2E-01 | 9.0E-01 | 7.4E-01 | 1.8E-01 |
| **Thr** | 2.9E-01 | 5.5E-01 | -1.7E-01 | 1.4E-01 | 6.8E-01 |  | 5.5E-01 | 8.5E-01 | 7.4E-01 | 8.3E-01 | 7.9E-01 | 7.9E-01 | 9.8E-01 | 8.6E-01 | 7.4E-01 | 4.7E-01 | 1.4E-01 | 3.0E-01 | 5.3E-01 | 1.0E+00 | 6.1E-01 | 9.1E-01 |
| **Trp** | 5.9E-01 | 6.4E-01 | 4.9E-01 | 5.2E-01 | 4.1E-01 | 1.9E-01 |  | 8.9E-01 | 5.2E-02 | 8.3E-01 | 2.6E-01 | 2.9E-01 | 3.3E-01 | 3.3E-01 | 2.2E-01 | 8.6E-01 | 2.2E-01 | 1.6E-01 | 5.0E-01 | 2.4E-01 | 5.3E-01 | 5.1E-01 |
| **ADMA** | -1.6E-01 | -4.2E-01 | 2.8E-01 | 2.5E-01 | 4.2E-01 | -6.2E-02 | 4.4E-02 |  | 3.7E-01 | 4.2E-01 | 4.2E-03 | 2.3E-03 | 2.4E-03 | 3.6E-04 | 2.0E-03 | 1.0E-02 | 3.1E-01 | 8.3E-01 | 5.7E-01 | 8.3E-01 | 8.1E-01 | 4.5E-04 |
| **Ac-Orn** | 2.4E-01 | 2.6E-01 | 4.4E-01 | 5.2E-01 | 4.3E-01 | -1.1E-01 | 5.7E-01 | 2.9E-01 |  | 3.7E-01 | 1.9E-01 | 1.6E-01 | 5.4E-01 | 1.9E-01 | 3.0E-01 | 7.6E-01 | 8.9E-02 | 1.2E-01 | 3.2E-01 | 7.7E-01 | 1.0E+00 | 1.7E-01 |
| **Kynurenine** | -1.3E-01 | -6.4E-02 | 4.9E-02 | -8.6E-02 | 1.5E-01 | -6.8E-02 | -7.1E-02 | 2.6E-01 | -2.8E-01 |  | 6.3E-01 | 5.7E-01 | 5.8E-01 | 6.7E-01 | 7.4E-01 | 4.9E-01 | 5.8E-01 | 2.9E-01 | 6.2E-01 | 2.4E-01 | 1.8E-01 | 8.9E-01 |
| **PC aa C36:4** | -2.2E-02 | 8.6E-02 | 5.0E-01 | 5.7E-01 | 4.4E-01 | 8.7E-02 | 3.6E-01 | 7.6E-01 | 4.1E-01 | 1.5E-01 |  | 2.9E-07 | 5.8E-06 | 5.7E-04 | 3.0E-04 | 2.6E-02 | 7.2E-02 | 4.2E-01 | 2.9E-01 | 3.2E-01 | 3.2E-01 | 7.1E-07 |
| **PC aa C36:5** | -5.2E-02 | 5.5E-02 | 4.0E-01 | 4.4E-01 | 4.9E-01 | 8.6E-02 | 3.4E-01 | 7.9E-01 | 4.3E-01 | 1.8E-01 | 9.7E-01 |  | 6.9E-05 | 4.9E-04 | 1.3E-03 | 8.1E-02 | 6.4E-02 | 3.7E-01 | 4.3E-01 | 5.4E-01 | 5.2E-01 | 3.0E-06 |
| **PC aa C38:5** | -1.1E-01 | -5.9E-02 | 5.1E-01 | 4.3E-01 | 2.8E-01 | -9.2E-03 | 3.1E-01 | 7.9E-01 | 2.0E-01 | 1.8E-01 | 9.4E-01 | 9.0E-01 |  | 7.2E-04 | 1.4E-04 | 7.1E-03 | 2.5E-01 | 4.0E-01 | 1.6E-01 | 2.7E-01 | 3.8E-01 | 6.4E-05 |
| **PC ae C40:1** | -1.5E-01 | -3.5E-02 | 5.2E-01 | 5.5E-01 | 5.2E-01 | 5.9E-02 | 3.1E-01 | 8.6E-01 | 4.1E-01 | 1.4E-01 | 8.4E-01 | 8.5E-01 | 8.4E-01 |  | 1.5E-06 | 2.6E-03 | 2.0E-02 | 2.4E-01 | 5.0E-01 | 3.2E-01 | 6.6E-01 | 3.6E-04 |
| **PC ae C42:2** | -3.2E-02 | 5.3E-02 | 5.4E-01 | 6.6E-01 | 4.6E-01 | 1.1E-01 | 3.8E-01 | 7.9E-01 | 3.2E-01 | 1.1E-01 | 8.6E-01 | 8.1E-01 | 8.8E-01 | 9.5E-01 |  | 6.3E-04 | 5.5E-02 | 2.3E-01 | 4.1E-01 | 1.7E-01 | 4.5E-01 | 5.1E-04 |
| **PC ae C44:3** | -2.6E-01 | -2.6E-01 | 5.8E-01 | 5.5E-01 | 9.9E-02 | -2.3E-01 | 5.9E-02 | 7.1E-01 | 9.7E-02 | 2.2E-01 | 6.4E-01 | 5.2E-01 | 7.3E-01 | 7.8E-01 | 8.4E-01 |  | 3.9E-01 | 4.8E-01 | 2.2E-01 | 2.0E-01 | 6.5E-01 | 2.4E-02 |
| **lysoPC a C14:0** | 5.5E-02 | 4.0E-01 | 4.1E-01 | 5.7E-01 | 6.9E-01 | 4.5E-01 | 3.8E-01 | 3.2E-01 | 5.1E-01 | -1.8E-01 | 5.4E-01 | 5.5E-01 | 3.6E-01 | 6.6E-01 | 5.7E-01 | 2.7E-01 |  | 4.6E-01 | 7.0E-01 | 1.8E-01 | 1.8E-01 | 8.1E-02 |
| **lysoPC a C20:3** | -9.6E-02 | 3.0E-01 | 2.7E-01 | 3.2E-01 | -1.3E-01 | -3.3E-01 | 4.4E-01 | 7.1E-02 | 4.7E-01 | -3.3E-01 | 2.6E-01 | 2.8E-01 | 2.7E-01 | 3.7E-01 | 3.7E-01 | 2.2E-01 | 2.4E-01 |  | 8.7E-01 | 5.8E-01 | 8.2E-01 | 5.2E-01 |
| **SM (OH) C14:1** | -6.8E-02 | -3.9E-01 | 8.0E-02 | 3.5E-02 | -3.8E-01 | -2.0E-01 | -2.2E-01 | 1.8E-01 | -3.1E-01 | -1.6E-01 | 3.3E-01 | 2.5E-01 | 4.4E-01 | 2.2E-01 | 2.6E-01 | 3.8E-01 | 1.3E-01 | -5.2E-02 |  | 1.2E-02 | 3.2E-03 | 2.2E-01 |
| **SM (OH) C16:1** | 4.1E-01 | 4.3E-02 | 3.5E-01 | 5.3E-01 | -4.2E-02 | 1.4E-03 | 3.6E-01 | 6.8E-02 | 9.4E-02 | -3.6E-01 | 3.1E-01 | 2.0E-01 | 3.4E-01 | 3.2E-01 | 4.3E-01 | 4.0E-01 | 4.2E-01 | 1.8E-01 | 7.0E-01 |  | 2.1E-04 | 3.1E-01 |
| **SM (OH) C22:2** | 4.0E-01 | 1.4E-01 | 8.8E-02 | 3.7E-01 | -1.1E-01 | 1.6E-01 | 2.0E-01 | -7.6E-02 | 8.3E-04 | -4.1E-01 | 3.1E-01 | 2.1E-01 | 2.8E-01 | 1.4E-01 | 2.4E-01 | 1.4E-01 | 4.1E-01 | 7.2E-02 | 7.7E-01 | 8.7E-01 |  | 3.2E-01 |
| **SM C22:3** | -3.1E-02 | -1.2E-01 | 3.9E-01 | 5.0E-01 | 4.2E-01 | 3.8E-02 | 2.1E-01 | 8.5E-01 | 4.2E-01 | 4.4E-02 | 9.6E-01 | 9.5E-01 | 9.0E-01 | 8.6E-01 | 8.5E-01 | 6.4E-01 | 5.2E-01 | 2.1E-01 | 3.8E-01 | 3.2E-01 | 3.2E-01 |  |

**Table S5**. Pearson correlation coefficients (lower triangle) and corresponding *p*-values (upper triangle) for the 22 metabolites identified by Boruta in pairwise comparisons of Italian Large White pigs.

|  | **C18:2** | **C3-DC (C4-OH)** | **C6 (C4:1-DC)** | **C6:1** | **Pro** | **Thr** | **Trp** | **ADMA** | **Ac-Orn** | **Kynurenine** | **PC aa C36:4** | **PC aa C36:5** | **PC aa C38:5** | **PC ae C40:1** | **PC ae C42:2** | **PC ae C44:3** | **lysoPC a C14:0** | **lysoPC a C20:3** | **SM (OH) C14:1** | **SM (OH) C16:1** | **SM (OH) C22:2** | **SM C22:3** |
| --- | --- | --- | --- | --- | --- | --- | --- | --- | --- | --- | --- | --- | --- | --- | --- | --- | --- | --- | --- | --- | --- | --- |
| **C18:2** |  | 7.0E-01 | 3.2E-01 | 9.6E-01 | 4.0E-01 | 8.4E-02 | 5.1E-01 | 1.1E-01 | 7.7E-01 | 1.7E-01 | 1.2E-01 | 1.7E-01 | 2.0E-01 | 1.1E-01 | 7.8E-01 | 8.6E-01 | 5.3E-01 | 5.1E-02 | 3.5E-01 | 3.2E-01 | 7.8E-02 | 8.3E-02 |
| **C3-DC (C4-OH)** | 1.3E-01 |  | 1.7E-01 | 1.5E-02 | 4.4E-03 | 3.9E-02 | 8.1E-02 | 4.7E-01 | 8.4E-01 | 9.6E-01 | 1.3E-02 | 5.1E-02 | 1.1E-02 | 6.7E-03 | 3.1E-03 | 2.3E-01 | 4.5E-01 | 5.6E-01 | 2.8E-01 | 3.8E-02 | 4.1E-01 | 3.4E-02 |
| **C6 (C4:1-DC)** | -3.1E-01 | 4.3E-01 |  | 4.7E-02 | 3.2E-01 | 8.5E-01 | 9.9E-01 | 8.2E-01 | 1.5E-01 | 4.4E-01 | 7.9E-01 | 5.5E-01 | 5.9E-01 | 9.8E-01 | 2.1E-01 | 4.3E-01 | 9.9E-01 | 3.8E-01 | 4.0E-01 | 1.3E-01 | 7.7E-01 | 6.2E-01 |
| **C6:1** | 1.6E-02 | 6.8E-01 | 5.8E-01 |  | 1.3E-01 | 7.5E-01 | 4.6E-01 | 7.4E-01 | 5.7E-01 | 5.1E-01 | 3.9E-01 | 5.8E-01 | 4.4E-01 | 2.2E-01 | 3.9E-01 | 5.4E-01 | 3.9E-01 | 8.7E-01 | 5.2E-01 | 2.3E-01 | 6.8E-01 | 4.5E-01 |
| **Pro** | -2.7E-01 | 7.6E-01 | 3.2E-01 | 4.6E-01 |  | 1.2E-01 | 6.1E-01 | 4.5E-01 | 6.2E-01 | 1.5E-01 | 2.8E-01 | 3.4E-01 | 2.4E-01 | 2.1E-01 | 3.6E-02 | 8.7E-02 | 2.6E-01 | 9.9E-01 | 6.3E-01 | 6.2E-01 | 5.4E-01 | 4.4E-01 |
| **Thr** | 5.2E-01 | 6.0E-01 | -6.0E-02 | 1.0E-01 | 4.7E-01 |  | 8.7E-02 | 5.9E-02 | 6.8E-01 | 7.8E-01 | 5.6E-02 | 1.4E-02 | 4.9E-02 | 1.6E-02 | 2.6E-02 | 5.2E-01 | 2.1E-01 | 3.0E-02 | 7.2E-01 | 3.9E-01 | 8.2E-01 | 8.7E-02 |
| **Trp** | 2.1E-01 | 5.2E-01 | 5.2E-03 | 2.3E-01 | 1.6E-01 | 5.1E-01 |  | 3.2E-01 | 2.9E-01 | 2.1E-01 | 7.1E-02 | 3.3E-02 | 2.3E-01 | 3.9E-02 | 1.1E-01 | 8.1E-01 | 3.3E-01 | 2.9E-01 | 3.0E-02 | 9.1E-02 | 1.8E-01 | 6.5E-02 |
| **ADMA** | 4.8E-01 | 2.3E-01 | -7.5E-02 | 1.1E-01 | 2.4E-01 | 5.6E-01 | 3.1E-01 |  | 6.6E-01 | 6.8E-01 | 8.5E-01 | 8.5E-01 | 9.8E-01 | 4.3E-01 | 6.8E-01 | 8.4E-01 | 2.7E-01 | 4.9E-01 | 7.6E-01 | 5.8E-01 | 8.9E-01 | 8.9E-01 |
| **Ac-Orn** | -9.4E-02 | 6.6E-02 | 4.4E-01 | 1.8E-01 | 1.6E-01 | 1.3E-01 | -3.3E-01 | -1.4E-01 |  | 7.1E-01 | 5.5E-01 | 9.6E-01 | 8.2E-01 | 8.3E-01 | 5.1E-01 | 3.3E-01 | 2.7E-01 | 9.6E-01 | 1.8E-01 | 6.0E-01 | 1.2E-01 | 4.3E-01 |
| **Kynurenine** | 4.2E-01 | -1.5E-02 | 2.5E-01 | 2.1E-01 | -4.4E-01 | -9.1E-02 | 3.9E-01 | 1.3E-01 | -1.2E-01 |  | 5.5E-01 | 7.0E-01 | 7.1E-01 | 4.2E-01 | 7.8E-01 | 5.7E-01 | 9.4E-02 | 6.4E-01 | 5.4E-03 | 3.0E-02 | 1.8E-02 | 3.8E-01 |
| **PC aa C36:4** | 4.7E-01 | 6.9E-01 | -8.6E-02 | 2.7E-01 | 3.4E-01 | 5.6E-01 | 5.4E-01 | 6.1E-02 | -1.9E-01 | 1.9E-01 |  | 5.8E-05 | 5.7E-05 | 6.5E-04 | 9.8E-03 | 2.7E-01 | 9.4E-01 | 1.5E-01 | 8.5E-02 | 5.8E-02 | 1.5E-02 | 2.4E-09 |
| **PC aa C36:5** | 4.2E-01 | 5.7E-01 | -1.9E-01 | 1.8E-01 | 3.0E-01 | 6.8E-01 | 6.2E-01 | 6.0E-02 | -1.6E-02 | 1.2E-01 | 9.0E-01 |  | 3.7E-03 | 5.4E-04 | 1.2E-02 | 2.8E-01 | 8.1E-01 | 3.0E-02 | 2.3E-01 | 2.6E-01 | 1.1E-01 | 8.2E-05 |
| **PC aa C38:5** | 4.0E-01 | 7.0E-01 | 1.7E-01 | 2.4E-01 | 3.7E-01 | 5.8E-01 | 3.7E-01 | 7.1E-03 | -7.2E-02 | 1.2E-01 | 9.0E-01 | 7.7E-01 |  | 1.4E-02 | 3.0E-03 | 3.8E-01 | 7.8E-01 | 2.3E-01 | 9.1E-02 | 3.8E-02 | 6.3E-02 | 4.0E-04 |
| **PC ae C40:1** | 4.9E-01 | 7.3E-01 | 8.5E-03 | 3.8E-01 | 3.9E-01 | 6.7E-01 | 6.0E-01 | 2.5E-01 | 6.9E-02 | 2.6E-01 | 8.4E-01 | 8.5E-01 | 6.8E-01 |  | 2.9E-03 | 5.8E-02 | 5.6E-01 | 3.7E-02 | 1.1E-01 | 2.6E-02 | 4.1E-02 | 7.7E-04 |
| **PC ae C42:2** | 9.2E-02 | 7.7E-01 | 3.9E-01 | 2.7E-01 | 6.1E-01 | 6.4E-01 | 4.8E-01 | 1.3E-01 | 2.1E-01 | 8.9E-02 | 7.1E-01 | 6.9E-01 | 7.8E-01 | 7.8E-01 |  | 2.2E-02 | 7.1E-01 | 2.9E-01 | 1.2E-01 | 1.9E-02 | 2.6E-01 | 2.5E-02 |
| **PC ae C44:3** | -5.8E-02 | 3.8E-01 | 2.5E-01 | 2.0E-01 | 5.1E-01 | 2.0E-01 | 7.8E-02 | 6.5E-02 | 3.1E-01 | 1.8E-01 | 3.5E-01 | 3.4E-01 | 2.8E-01 | 5.6E-01 | 6.5E-01 |  | 9.6E-01 | 5.9E-01 | 5.4E-01 | 1.3E-01 | 3.5E-01 | 2.6E-01 |
| **lysoPC a C14:0** | 2.0E-01 | 2.4E-01 | -5.0E-03 | 2.8E-01 | 3.6E-01 | 3.9E-01 | -3.1E-01 | 3.4E-01 | 3.5E-01 | -5.0E-01 | 2.3E-02 | 7.8E-02 | 9.2E-02 | 1.9E-01 | 1.2E-01 | -1.8E-02 |  | 2.5E-01 | 2.2E-01 | 5.0E-01 | 3.6E-01 | 8.6E-01 |
| **lysoPC a C20:3** | 5.7E-01 | 1.9E-01 | -2.8E-01 | 5.2E-02 | -5.3E-03 | 6.2E-01 | 3.3E-01 | 2.2E-01 | -1.8E-02 | 1.5E-01 | 4.4E-01 | 6.3E-01 | 3.8E-01 | 6.1E-01 | 3.3E-01 | 1.7E-01 | 3.6E-01 |  | 3.9E-01 | 4.5E-01 | 2.5E-01 | 1.1E-01 |
| **SM (OH) C14:1** | 3.0E-01 | 3.4E-01 | 2.7E-01 | 2.1E-01 | -1.5E-01 | 1.2E-01 | 6.3E-01 | 9.8E-02 | -4.1E-01 | 7.5E-01 | 5.2E-01 | 3.7E-01 | 5.1E-01 | 4.9E-01 | 4.8E-01 | 2.0E-01 | -3.8E-01 | 2.7E-01 |  | 2.2E-04 | 6.9E-04 | 6.3E-02 |
| **SM (OH) C16:1** | 3.2E-01 | 6.0E-01 | 4.6E-01 | 3.7E-01 | 1.6E-01 | 2.8E-01 | 5.1E-01 | 1.8E-01 | -1.7E-01 | 6.2E-01 | 5.6E-01 | 3.5E-01 | 6.0E-01 | 6.4E-01 | 6.6E-01 | 4.6E-01 | -2.1E-01 | 2.4E-01 | 8.7E-01 |  | 4.2E-03 | 5.3E-02 |
| **SM (OH) C22:2** | 5.3E-01 | 2.6E-01 | -9.4E-02 | 1.3E-01 | -1.9E-01 | 7.5E-02 | 4.1E-01 | 4.3E-02 | -4.7E-01 | 6.7E-01 | 6.8E-01 | 4.8E-01 | 5.5E-01 | 6.0E-01 | 3.5E-01 | 2.9E-01 | -2.9E-01 | 3.6E-01 | 8.4E-01 | 7.6E-01 |  | 5.0E-03 |
| **SM C22:3** | 5.2E-01 | 6.1E-01 | -1.6E-01 | 2.4E-01 | 2.4E-01 | 5.1E-01 | 5.5E-01 | 4.5E-02 | -2.5E-01 | 2.8E-01 | 9.9E-01 | 9.0E-01 | 8.5E-01 | 8.3E-01 | 6.4E-01 | 3.5E-01 | -5.9E-02 | 4.8E-01 | 5.5E-01 | 5.7E-01 | 7.5E-01 |  |

**Table S6**. Percentage (%) of Boruta runs in which each of the six metabolites was selected in the Boruta-based reanalysis of the 2016 Italian Large White and Italian Duroc dataset [10], along with Mean Decrease Gini (MDG) values from the random forest model.

|  |  |  | **Plasma** | | **Serum** | |
| --- | --- | --- | --- | --- | --- | --- |
| **Metabolite** | **Biochemical name** | **Metabolite class** | **Runs (%)** | **MDG** | **Runs (%)** | **MDG** |
| Ac-Orn | Acetylornithine | Biogenic amine | 100 | 0.17 | 100 | 0.20 |
| Kynurenine | Kynurenine | Biogenic amine | 98 | 0.11 | - | - |
| SM (OH) C14:1 | Hydroxysphingomyeline C14:1 | Sphingomyelin | 95 | 0.10 | 97 | 0.10 |
| SM (OH) C16:1 | Hydroxysphingomyeline C16:1 | Sphingomyelin | 97 | 0.10 | - | - |
| SM C16:0 | Sphingomyeline C16:0 | Sphingomyelin | - |  | 93 | 0.09 |
| SM C16:1 | Sphingomyeline C16:1 | Sphingomyelin | - |  | 96 | 0.09 |
